# Environmentally Selected Aphid Variants in Clonality Context Display Differential Patterns of Methylation in the Genome

**DOI:** 10.1371/journal.pone.0115022

**Published:** 2014-12-31

**Authors:** Claude Pasquier, Mathilde Clément, Aviv Dombrovsky, Stéphanie Penaud, Martine Da Rocha, Corinne Rancurel, Neil Ledger, Maria Capovilla, Alain Robichon

**Affiliations:** 1 Institute Sophia Agrobiotech, INRA/CNRS/UNS, University Nice Sophia Antipolis, Sophia Antipolis, France; 2 Institute of Plant Protection, Volcani Center, Rehovot, Israel; 3 Institute of Developmental Biology and Cancer, CNRS, University Nice Sophia Antipolis, Sophia Antipolis, France; 4 Beckman Coulter Genomics SA, Grenoble, France; Universität Stuttgart, Germany

## Abstract

Heritability of acquired phenotypic traits is an adaptive evolutionary process that appears more complex than the basic allele selection guided by environmental pressure. In insects, the trans-generational transmission of epigenetic marks in clonal and/or sexual species is poorly documented. Aphids were used as a model to explore this feature because their asexual phase generates a stochastic and/or environment-oriented repertoire of variants. The *a priori* unchanged genome in clonal individuals prompts us to hypothesize whether covalent methyl DNA marks might be associated to the phenotypic variability and fitness selection. The full differential transcriptome between two environmentally selected clonal variants that originated from the same founder mother was mapped on the entire genomic scaffolds, in parallel with the methyl cytosine distribution. Data suggest that the assortments of heavily methylated DNA sites are distinct in these two clonal phenotypes. This might constitute an epigenetic mechanism that confers the robust adaptation of insect species to various environments involving clonal reproduction.

## Introduction

In most species, epigenetic marks on DNA are partly related to environment-dependent covalent binding of a methyl group to cytosine and it has been commonly accepted that this chemical modification initiates chromatin remodeling and changes in the regulation of gene expression [1]. The mapping of the methyl marks on the genome has been examined in various models such as the flowering plant *Arabidopsis thaliana* [2]–[9] and the honeybee *Apis mellifera* [10]–[14]. This epigenetic signaling is currently under scrutiny because in the past many parallel observations related to different species have suggested that some environment-dependent epigenetic marks are heritable [15]–[18]. The paradigm broadly accepted is that the epigenetic modifications that are propagated across a variable number of generations orchestrate a flexible heredity of some advantageous phenotypic traits [16], [17], [19], [20]. The conditions of heritability of methyl cytosine residues come from the fact that the methylase(s) bind to a methylated CpG motif present in one strand of DNA and then methylate the opposite site of the daughter strand during replication [21].

These phenomena are poorly documented in insects. Thus, the aim of this report consists in investigating how some epigenetic marks might be attributed specifically to genomic sequences in an heritable phenotype that has been selected in an environmental context. The model used in this work is the aphid *Acyrthosiphon pisum*. This species is clonal during spring and summer, but the combination of shorter photoperiodicity and cold temperature in fall triggers the appearance of male and female sexual animals [22]–[24]. Moreover, aphids carry primary endosymbiont bacteria (*Buchnera aphidicola*) that supply the aphids with essential compounds like amino acids [25]–[27]. The secondary endosymbionts are facultative, but endow the aphid host with properties like resistance to pathogens [28] or green pigmentation [29].

In contrast to the common thought that equates clonality with molecular and genetic identity, we have shown that clonal reproduction in the insect model *A. pisum* is a powerful mechanism to create a repertoire of variants with distinct behavioral and physiological traits [30]. As an example, the aphid genome along with that of plants, algae and some fungi amazingly contains the genes able to synthesize carotene molecules, but in aphids carotenoid synthesis seems strictly regulated by environmental factors [31], [32]. To this regard, we have observed that the synthesis of pigments in a given aphid population is a density- and frequency-dependent phenomenon: optimal conditions trigger a strong carotene synthesis (*orange* aphids), a high population-density leads to the arrest of carotene synthesis in a proportion of individuals increasing with time (*white* aphids), whereas cold temperatures produce a green pigmentation (*green* aphids) [23], [30]. We have shown that *white* aphids can also be obtained by treating parthenogenetic *orange* aphids with inhibitors of DNA methyl transferases [30]. Many sites in this white variant genome were hypo methylated (whereas they were densely methylated in orange aphids) and the morph distribution was drastically modified with the quasi disappearance of the winged aphids between generations 5 to 10. Each of these variants (orange and white) can generate the other phenotype. These phenotypes are therefore inter-convertible under the pressure of environment in progenies (these phenotypic traits are acquired for their life span and never seen in constant environmental conditions), but not in the founder mother.

Modalities to shape clonal phenotypic variants produced without sex, and consequently without gene mixing by crossing over in meiosis, are still poorly understood. Our assumption is that this scenario appears to limit the role of allele recruitment and chromosome recombination that sexuality renders possible. This phenotypic repertoire in conditions where the genome is apparently unchanged was analyzed to determine whether some variants are correlated with epigenetic marks located on specific sites in the genomic scaffolds. For this purpose, covalent modification by addition of methyl groups on the whole aphid genome was investigated as the epigenetic mark that is the most amenable to analytical procedures. In order to address the epigenetic hypothesis as an alternative and/or parallel scenario to allele selection, we carried out a high throughput analysis of DNA methylation to investigate how the heavily methylated zones in the aphid genome vary between environment-dependent variants. We performed an extensive analysis of DNA fragments enriched in methyl CpG motifs in two environmentally selected variants originated from a unique aphid parthenogenetic founder mother: the *orange* (22°C adapted) and the *green* (8°C adapted). In addition, we document the full transcriptomic differences between the two aphid variants. The differential expression of extensive gene networks has been analyzed in relation to the density of DNA methylation in/around genes for these two clonal variants.

## Results

### Selection of an aphid variant with a singular pigmentation

**Figure 1:**
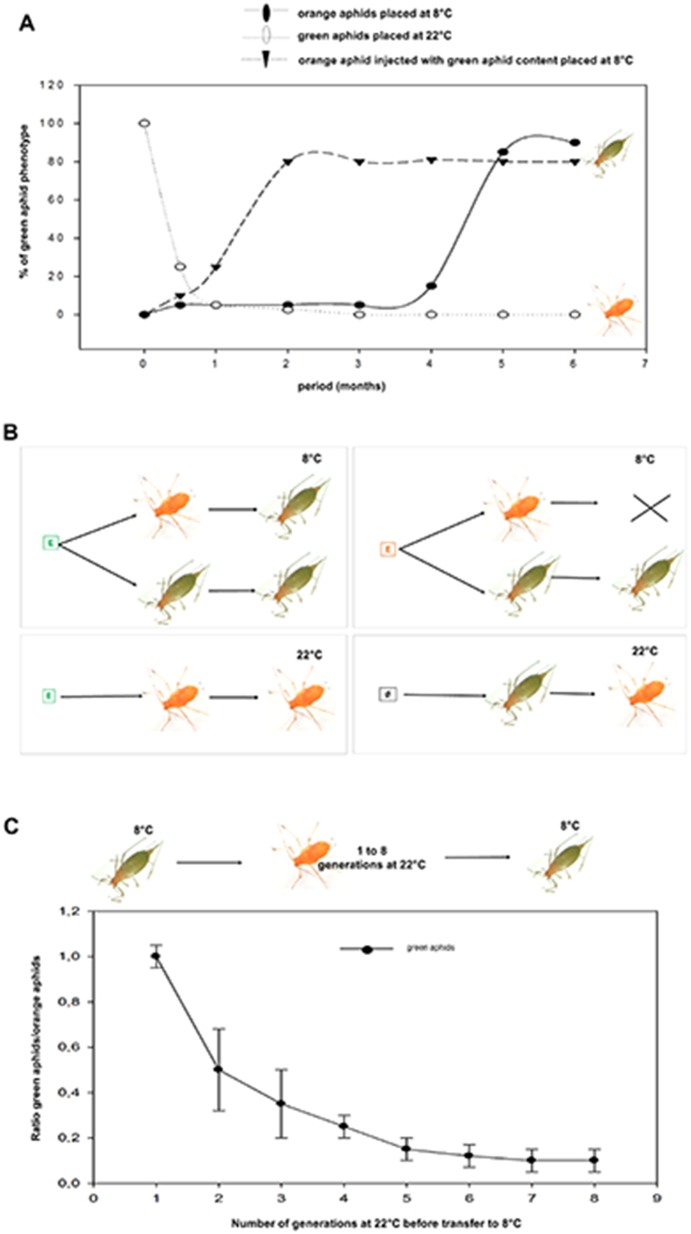
Selection of the green variant. (A) The scheme summarizes the results of experiments using injection of aphid extracts adapted to 8°C or to 22°C in aphid mothers adapted to 8°C (green squares) or to 22°C (red squares). The empty box represents injection of buffer (Ringer solution) only. Arrows show the passage *orange* to *green* or *vice versa*. The cross indicates the death of progenies. (B) Ten orange adult aphids reared at 22°C from a single founder were placed at 8°C each day (X-axis) before the emergence of a *green* phenotype (black circles) after 5 months. *Orange* aphids injected with *green* aphid extract gave immediately *green* progenies in cold (black triangles). *Green* aphids placed at 22°C lost their green pigments after few days and their progenies were immediately orange (open circles). (C) *Green* aphids were placed at 22°C for 8 generations and for each one, 20 newly *orange* adults were placed back at 8°C. A preliminary experiment allowed us to determine the time required to get 20 newly *green* adult aphids from the 20 original orange at the first generation. This time scale was used to count the newly *green* adult aphids at each generation and data are represented as the ratio newly *green* adults versus initial *orange* aphids. Dots are the mean+/− SEM, n = 4.

Clonal individuals from the same *orange* founder mother were propagated separately at different temperature conditions. Ten *orange* parthenogenetic adult aphids were placed each day at 8°C, conditions at which progenies did not survive with most of the larvae dying between stage 2 and stage 4. After five months, a viable and robust colony of *green* variants suddenly emerged (Fig. 1A), characterized by a longer life cycle and slightly bigger adult body size (data not shown). Thus, the *orange* descendants of a single founder mother that are adapted to 22°C have the potential to generate a cold-adapted lineage, viable and heritable at the conditions that allowed us to select it. Interestingly, the *green* mothers placed back at 22°C experienced a demographic fall for few generations and progenies were always orange (Fig. 1A). Thus, a clonal aphid population submitted to environmental pressure leads to adapted variants. The fastness/efficiency of the selection process strongly suggest that selection is under epigenetic control. To further explore this scenario we performed two types of experiments: the first, consists in injecting crude soluble extract of *green* aphids into the abdomen of adult *orange* aphids, which were then placed at 8°C (Fig. 1B). The first generation of progenies was immediately green and viable, bypassing the long and fastidious selection process. The newly emerged *green* variant obtained in these conditions gave a robust heritable lineage in cold conditions (Fig. 1A and 1B). On the contrary, the injection of this *green* extract in *orange* mothers placed at 22°C failed to produce the *green* variants (Fig. 1B). Overall, this argues in favor of maternal transmissible factors that might guide the phenotypic fates of the progenies. In the second experiment, the conditions to reverse phenotypes have been analyzed to determine whether a memory effect might facilitate the process. Green aphids grown at 22°C for 1–8 generations become *orange* and a sampling of each generation was placed back at 8°C. After one generation at 22°C, all progenies became green again when placed back at 8°C, but after each subsequent generation at 22°C, only a decreasing proportion of them were able to generate green progenies at 8°C (Fig. 1C). These results show that aphids originated from a lost phenotype/environment (*green*) are able to generate descendants presenting the traits of the ancestral phenotype (*orange*) in a facilitated way when placed back in initial conditions. However, this phenotypic memory vanishes quickly after few generations (Fig. 1C).

### High throughput analysis of the methylome in the two distinct aphid clonal variants originated from the same founder mother

The methylome of two aphid variants (*orange* and *green*) originated from the same *orange* founder mother was determined on the hypothesis that DNA methylation is a likely molecular mechanism that orchestrates the clonal phenotypic repertoire. We performed the specific pull-down of heavily methylated DNA fragments using an engineered transcriptional repressor (MBD2) with a strong affinity for the methyl CG (Methyl collector, *Active Motif*). The steps include the enzymatic cleavage of aphid genomic DNA, the pull-down of methyl fragments, the construction of libraries with these fragments, their subsequent pyrosequencing and finally a procedure to quantify reads. These reads correspond to strictly identical and/or overlapping or unique sequences (see Experimental Procedures for details). Each individual read for both variants was matched on the full genome and the visualization of each decorated scaffold allows us to examine the increase/decrease of methylation in many different loci in one or the other variant in parallel with the unchanged methylated sites as internal control (see Experimental and S1 Procedures). This strategy was chosen instead of alternative methods using the anti-5-methylcytosine antibody or the high throughput bisulfite DNA sequencing due to their technical limits (see S1 Procedures for arguments).

**Figure 2:**
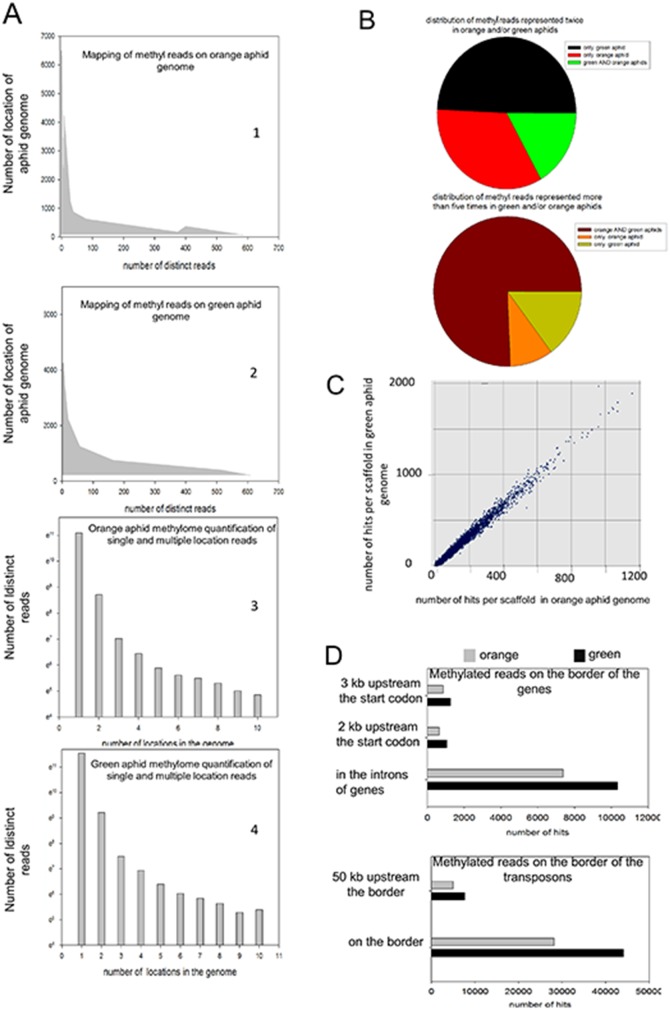
Comparative analysis of the methylome hits matching to genome, genes and transposons between the *orange* and *green* variants. (A) Several hundreds of distinct reads found in both variants map on a large number of locations in scaffolds. Two graphs represent the number of unique reads (X axis) corresponding to the number of genomic locations retrieved by exact match (Y axis) (A1 and A2). The 50 reads the most represented in either variant totalize 107,258 locations for the *orange* and 118,966 for the *green*, which provides an internal control for further comparative analysis. On the other hand, the number of distinct reads that fits from 1 to 10 locations in the genome by exact match is plotted for both variants. About 65,615 and 103,529 unique reads match one location; 6,094 and 10,078 match two locations; 361 and 603, 5 locations for the *orange* and the *green*, respectively. (A3 and 4) (B) Distribution of methyl reads represented twice only in *green* (7,735), *orange* (5,339) and one in either variants (2,639). The same analysis was performed with methyl reads represented more than 5 times in both variants (917) exclusively in the *green* (181) or the *orange* (115). (C) The methyl fragments reads obtained with the *orange* and the *green* samples were mapped on scaffolds. X and Y-axis correspond to the number of hits per scaffold for the *orange* and the *green* aphids, respectively. Coefficient of correlation r = 0.995. (D) Comparative number of hits in introns, in promoters (2 and 3 kb upstream), border of transposons and arbitrary at 50 kb (49–51 kb) upstream of transposons.

The total reads of the methylome were matched on the scaffolds. Distinct reads (as singleton or multiple exact copies of the same reads) were analyzed regarding the number of locations they were found in the genome. We reasoned that methyl reads matching on a large number of positions in the genome and present in both variants may constitute an internal control to assess our procedures. Few methyl reads in the *orange* and *green* aphids present a large number of exact matches on different locations of the full set of scaffolds (Fig. 2A1 and 2A2). Interestingly, one methyl read matches 6,424 genomic locations for the *orange* and is also found in *green* reads. About ten overlapping methyl reads that are found in the *green* and *orange* aphids are represented in thereabout 4,500 genomic locations. Eight methyl reads are found between 4,107 and 4,550 locations in the scaffolds for the *green* and *orange* phenotypes; 2,000 methyl reads for the *orange* and 3,342 reads for the *green* aphids match between 11 and 99 positions (Fig. 2A1 and 2A2). The 50 reads the most represented in either variant totalize 107,258 locations for the *orange* and 118,966 for the *green*, which provides an internal control for further comparative analysis. A scrutiny of this category of reads reveals that some of them present different sizes and overlap with others and this was observed in either variant. This suggests that genomic complexity is less extended than expected and some differences might partly come from the heterogeneity generated by enzymatic DNA cleavage. Table S1 present a collection of reads found in the *green* and the *orange* phenotypes that are among the 100 most represented in the genome.

The extension of the analysis uncovers that 65,000 different methyl reads for the *orange* and 103,000 for the *green* match only one location in each genome (Fig. 2A3 and 2A4). To further investigate the robustness of the method, the population of methyl reads found identically twice in pyrosequencing in one or the two variants was analyzed: 7,735 reads were found twice only in the *green* variant, 5,339 only in the *orange* and 2,639 once in each variant. Table S2 present the list of 21,000 reads and their sequence that are found at least twice in one or both variants. The population of methyl reads represented identically more than five times in pyrosequencing was also analyzed the same way: 917 reads were found in the *orange* and *green*, 115 only in the *orange* and 181 only in the *green* (Fig. 2B). Table S3 presents the list of 1200 reads and their sequences that are found at least five times in one or both variants.

A subset of methyl reads was randomly checked by the bisulfite sequencing technique (S1 Procedures). The comparative percentage of methyl groups *per* site into two retrieved genomic fragments is presented in S1 Fig. This allowed us to verify the robustness of the pull-down/pyrosequencing procedure and the reliability of the comparative data between the *orange* and *green* phenotypes.

The correlation between the *orange* and the *green* variants regarding the number of methylome hits on each scaffold gave a coefficient r = 0.99 (Fig. 2C). This strongly suggests that the methyl DNA regions are similar between variants and the differences partly involve variable assortments of methyl cytosines within conserved hot spots. The analysis of the mapping of reads on the totality of the genomic scaffolds seems to retrieve a slight global increase of methylation by +0.66 (log 2) in the *green* aphids. This was observed in all the sub regions of the genome like transposons, exons, introns or promoters (Fig. 2D). Moreover, the analysis of the most methylated scaffolds (calculated by the arithmetic mean of matched reads in both samples *versus* the size of the scaffolds) highlights that a large majority of them are more methylated in the *green* than in the *orange* variant. The methyl read densities are not uniformly distributed and appear concentrated in hot spots. To prolong this analysis we have quantified the level of methylation in each sub-class of genomic elements like transposons, genes and promoters: 16,758 methyl reads for the *orange* and 25,937 for the *green* aphids match transposons; 36,503 methyl reads for the *orange* and 58,174 for the *green* aphids match the border of transposons (1 kb upstream or downstream) whereas the regions at 50 kb upstream the border gave low level of reads with little variations between the two variants (5,500 and 6,700 for the *orange* and the *green*, respectively) (Fig. 2D). Regarding genes, 13,521 methyl reads in the *orange* and 19,377 in the *green* aphids match the body of genes whereas only 2,241 for the *orange* and 3,156 for the *green* aphids match their promoters (from 0 to 2 kb upstream). The number of methyl reads was 10,586 and 7,125 within the introns for the *green* and the *orange* respectively, whereas the methyl reads at 2 kb and 3 kb from the start of genes for both variants were not very different (850 and 1150 *versus* 810 and 980, respectively) (Fig. 2D). We undertook a rough comparison of methyl reads distribution between the two clonal variants, although all these determinations were not normalized by the ratio of CpG *per* DNA size unit.

### High throughput transcriptomic differences between the two distinct aphid clonal variants originated from the same founder mother

A differential transcriptomic analysis between these two clonal variants (*orange* and *green*) was carried out in order to assess the scale of gene expression modifications. The differences in gene expression were determined through a subtractive procedure of RNA enrichment. The suppression subtractive hybridization (SSH) that we have chosen allowed us to use the background/noise as an internal control to calibrate the samples for the following reason: the procedure does not subtract the linear amplification of cDNAs (internal control) in contrast to the exponentially amplified cDNAs corresponding to the subtracted RNA (see S1 Procedures).

**Figure 3:**
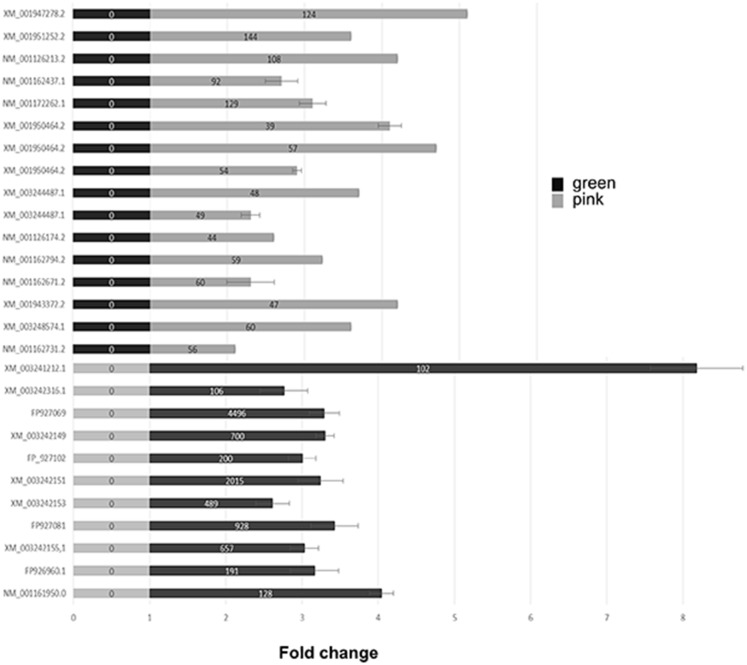
Quantitative PCR analysis of selected genes to assess the pyrosequencing transcriptomic data. Bars represent fold expression of gene of interest in the *orange* aphids (black) and the *green*-cold-adapted ones (grey). For each gene of interest, the normalized expression was rescaled by minimal sample value. The accumulation of each transcript was measured in three independent biological samples for each aphid variant. Statistically significant differences were determined by Student’s test analyses. The numbers in the bars are the reads obtained by RNA enrichment (SSH) followed by pyrosequencing of the corresponding cDNA.

The two cDNAs libraries corresponding to the *orange* or *green* variant were pyrosequenced (60,945 reads for the *orange* and 53,842 reads for the *green*). The reads were contigated and matched against the scaffolds. Out of 2,540 *orange* contigs and 2,008 *green* contigs, a reciprocal BLAST between the *green* and *orange* samples gave 864 pairs of contigs with high similarity (e-value <1e-50) and associated to a low number of reads. This category of contigs was used as internal control to calibrate the samples and then the contigs whose number of associated reads displays a large difference between the two variants were considered for further analysis (see Experimental and S1 Procedures for the cut off thresholds). The reliability of the data obtained with the SSH technique followed by pyrosequencing analysis was assessed by quantitative PCR. A subset of genes that were expressed in only one variant was selected arbitrarily and the analysis by qPCR is presented in Fig. 3. We observe a strong convergent trend between the data obtained with the two protocols. Although few genes retrieved by SSH technique gave little change in qPCR analysis, contradictory results were rarely obtained.

### Dual match of methyl CpG densities and differential transcriptomes

**Figure 4:**
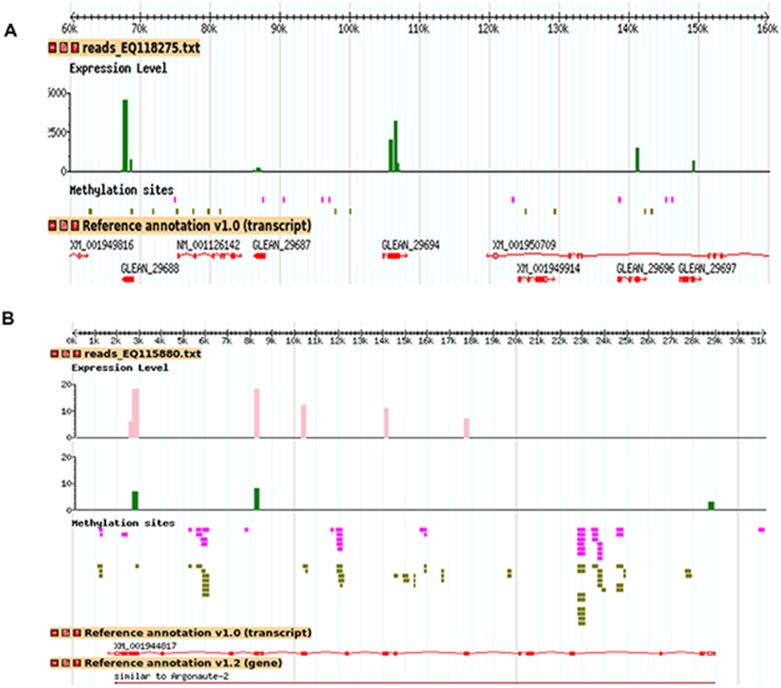
Representative scaffolds decorated with the mapping of the exact matches corresponding to the methyl reads and the transcriptomic contigs. The methylome reads were directly mapped to the genome scaffolds of *A. pisum* strain LSR1 version Acyr_1.0 with the program Razers [43]. The contigs were searched for sequence similarity using BLAST [46]. Vertical bars are the number of reads corresponding to the contigated fragments obtained after subtractive enrichment of transcripts (pink for *orange* aphids and green for *green* aphids). Each pink and green horizontal trait represents a methyl read that is at least represented twice in pyrosequencing in the *orange* and *green* aphids and the thick traits are overlapping or contiguous reads. The methyl reads were mapped on scaffolds disregarding the fact that some of them match the genome elsewhere. (A) One scaffold shows a strong gene expression of five genes in the *green* aphids, whereas these transcripts are absent in the *orange*. (B) Expression and methylation of one specific gene encoding *Argonaute-2*.

The public aphid base/NCBI resource allows us to construct a dual match: methylome and differential transcriptome on each individual scaffold (GBrowse). Two arbitrary decorated scaffolds are presented in Fig. 4A and 4B. One scaffold (Fig. 4A) seems unique in that it highlights a large number of over expressed hits for the *green* variant. Indeed, this scaffold (EQ118275) of 214,228 bp, corresponding to 0.05% of the total length of the assembled scaffolds, concentrates 13% of the over expressed hits for the *green* variant. These hits are concentrated on 5 computationally annotated and unknown genes (GLEAN_29688/ACYPI49688,GLEAN_29694/ACYPI006423,GLEAN_29696/ACYPI49696,GLEAN_29697/ACYPI49697 and GLEAN_29687/ACYPI49687) for which no reads were found in the *orange* variant (Fig. 4A). Moreover, the differential expression of some genes between the two phenotypes seems to affect specifically some exons (Fig. 4B). The first 500 most methylated scaffolds are all more methylated in the *green* and 475 of them (95%) gave a decrease of the number of transcriptomic reads in the *green* aphids. For the 10,000 most methylated scaffolds, this trend was largely attenuated, 8,463 were found equally or more methylated in the *green* whereas 8,847 (88.47%) gave a decrease of the number of the differentially expressed genes in the *green* aphids. If a direct causality of the DNA methylation and gene expression producing phenotypic plasticity appears difficult to apprehend (S2 Fig.), overall data suggest that specific patterns of DNA methylation regulating gene expression are associated to clonal phenotypes favored by environmental pressure.

### Gene Ontology term enrichment for genes showing modification of methylation

The Gene Ontology term enrichment (GO) provides a controlled vocabulary to describe (“annotate”) genes or gene products in three categories: biological process, molecular function and cellular component. Identification of over or under represented GO terms among a given list of genes is commonly used to better understand their integrated cross-talk. In this work, GO term enrichment analysis has been used to address the probability of gene network changes observed between the two clonal aphid variants originated from the same founder mother (*orange* and *green*). The procedure is detailed in the Experimental Procedures section and the extensive analysis regarding the strong increase and decrease of differentially expressed genes in the *green* variant compared to the *orange* is reported in S3-S6 Figs. and S1 Procedures. A gene was considered to have a strong variation of expression if 20 or more transcriptome reads are associated with this gene in one sample whereas no transcriptomic read is associated with the other sample. Some coherent trends emerge regarding the biological process: the genes associated to strong decrease of expression in the *green* are relevant to mRNA catabolic process, DNA-dependent replication, translation process, mRNA splicing and mRNA transport; the genes associated to a strong increase of expression in the *green* are related to aspartate/glutamate metabolism, phosphoinoside metabolism, lipid metabolic process, ATP synthesis coupled to electron transport, mitochondrial electron chain, lipid and carbohydrate transport and GTPase activity (S3-S6 Figs. and S1 Procedures). In order to address whether a range of regulation of the transcripts might be optimally associated with the methylation state of the corresponding genomic loci, two types of analysis were carried out.

First, the analysis of methylation was performed separately in the body of the genes and in their promoters (2 kb from the initiation of transcription). The category of genes with a moderate to high decrease of expression (> two fold less reads) was subdivided into minimally two fold increase or decrease of methyl reads in the gene body and/or the promoters. Inversely, the same protocol was used to analyze genes presenting a moderate to high increase of expression (> two fold more reads) and the correlation with their methylation variations (> two fold). A p-value was attributed to each term of the GO list and huge discrepancies were observed among them (S7-S14 Figs. and S1 Procedures). According to this scenario, a moderate to strong increase or decrease of numerous differentially expressed genes in the *green versus* the *orange* correlates well with the decrease (minimum ÷2) or the increase (minimum ×2) of methyl reads, respectively. However many other genes seem to vary in reverse order, which suggests that multiple modalities might be operating.

**Figure 5:**
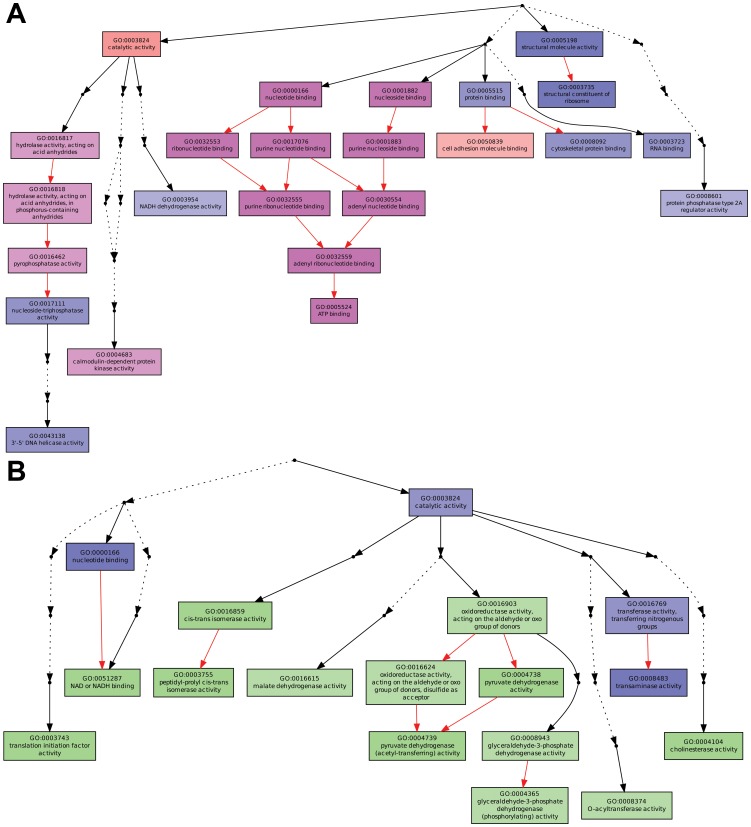
Gene Ontology term enrichment for genes showing a variation (>twofold) of methylation. Both diagrams display enriched GO terms and their hierarchical relationships with respect to the “Molecular Function” GO category. Boxes represent GO terms. (A) Significantly enriched GO terms that present a strong reduction of reads in the *green* compared to the *orange* aphids (using a p-value<0.05, see main text and S1 Procedures for the cut off threshold). Terms marked pink have a minimum two-fold increase of methylation, whereas terms marked blue do not show methylation differences. (B) Significantly enriched GO terms that present a strong increase of reads in the *green* compared to the *orange* aphids using a p-value<0.05 (see main text and S1 Procedures for the cut off threshold selection). Terms marked green have a minimum two-fold decrease of methylation, whereas terms marked blue do not show methylation differences. The degree of color saturation of each box is positively correlated with the significance of enrichment of the corresponding GO term. Dots represent omitted terms that are not significantly found. Edges (arrows) stand for connections between different GO terms. Red edges stand for relationships between two enriched GO terms, black solid edges for relationships between enriched and non enriched terms and black dashed edges for relationships between two un-enriched GO terms.

Second, we re-analyzed the list of genes showing strong increase or decrease of differentially expressed genes (at least 20 reads in a variant *versus* 0 in the other) associated with the methylation state (minimum two fold increase or decrease) and we proceeded by selecting the terms corresponding to a p-value cut off of <0.05 (Fig. 5). In this modality of analysis, we selected gene networks in which an increase of methylation in the *green* (in the gene body or in the promoter) was associated with a decrease of gene expression and oppositely a decrease of methylation was associated with an increase of gene expression (Fig. 5). However, some genes and/or gene networks did not correspond to this scenario, showing the opposite correlation between variation of methylation and expression (S7-S14 Figs. and S1 Procedures).

Altogether these results are summarized in table 1 along with a p-value attributed to each GO. In this table, a less stringent cut off was used for the Gene Ontology term enrichment analysis of genes showing minimally a twofold variation in reads (arbitrary minimal threshold of 20 reads, p-value<0.05) and for which an increase or decrease of methyl reads is associated. We observe that, under this analytical mode, many “Molecular Function” GO categories under- or over-expressed inversely correlate with their methylation state. The p-values for the under expressed genes associated to an increase of methylation appear more significant than the opposite trend (over expressed associated to decrease of methylation).

**Table 1:** Statistical analysis of the correlation GO term enrichment and methylation.

**A Molecular functions of over-expressed genes in the green variant *versus* the orange**		
GOID	Term name	p-value
GO:0000166	nucleotide binding	0.0015
GO:0003824	catalytic activity	0.0229
GO:0008483	transaminase activity	0.0013
GO:0016769	transferase activity, transferring nitrogenous groups	0.0089
**B Molecular functions of over-expressed genes in the green variant *versus* the orange with a decrease of methylation**		
GOID	Term name	p-value
GO:0003743	translation initiation factor activity	0.0265
GO:0003755	peptidyl-prolyl cis-trans isomerase activity	0.0191
GO:0004104	cholinesterase activity	0.0287
GO:0004365	glyceraldehyde-3-phosphate dehydrogenase (phosphorylating) activity	0.0392
GO:0004738	pyruvate dehydrogenase activity	0.0287
GO:0004739	pyruvate dehydrogenase (acetyl-transferring) activity	0.0287
GO:0008374	O-acyltransferase activity	0.0392
GO:0008943	glyceraldehyde-3-phosphate dehydrogenase activity	0.0392
GO:0016615	malate dehydrogenase activity	0.0499
GO:0016624	oxidoreductase activity, acting on the aldehyde or oxo group of donors, disulfide as acceptor	0.0499
GO:0016859	cis-trans isomerase activity	0.0190
GO:0016903	oxidoreductase activity, acting on the aldehyde or oxo group of donors	0.0394
GO:0051287	NAD or NADH binding	0.0191
**C Molecular functions of under-expressed genes in the green variant *versus* the orange**		
GOID	Term name	p-value
GO:0000166	nucleotide binding	7.29e-5
GO:0001882	nucleoside binding	0.0001
GO:0001883	purine nucleoside binding	0.0001
GO:0003723	RNA binding	0.0127
GO:0003735	structural constituent of ribosome	0.0009
GO:0003954	NADH dehydrogenase activity	0.0359
GO:0004683	calmodulin-dependent protein kinase activity	0.0341
GO:0005198	structural molecule activity	0.0019
GO:0005515	protein binding	0.0218
GO:0005524	ATP binding	8.55e-5
GO:0008092	cytoskeletal protein binding	0.0100
GO:0008601	protein phosphatase type 2A regulator activity	0.0477
GO:0016462	pyrophosphatase activity	0.0049
GO:0016817	hydrolase activity, acting on acid anhydrides	0.0043
GO:0016818	hydrolase activity, acting on acid anhydrides, in phosphorus-containing anhydrides	0.0063
GO:0017076	purine nucleotide binding	8.36e-5
GO:0017111	nucleoside-triphosphatase activity	0.0196
GO:0030554	adenyl nucleotide binding	0.0001
GO:0032553	ribonucleotide binding	6.28e-5
GO:0032555	purine ribonucleotide binding	6.28e-5
GO:0032559	adenyl ribonucleotide binding	0.0001
GO:0043138	3′-5′ DNA helicase activity	0.0063
**D Molecular functions of under-expressed genes with an increase of methylation**		
GOID	Term name	p-value
GO:0000166	nucleotide binding	0.0101
GO:0001882	nucleoside binding	0.0056
GO:0001883	purine nucleoside binding	0.0054
GO:0003824	catalytic activity	0.0231
GO:0004683	calmodulin-dependent protein kinase activity	0.0042
GO:0005524	ATP binding	0.0024
GO:0016462	pyrophosphatase activity	0.0167
GO:0016817	hydrolase activity, acting on acid anhydrides	0.0221
GO:0016818	hydrolase activity, acting on acid anhydrides, in phosphorus-containing anhydrides	0.0188
GO:0017076	purine nucleotide binding	0.0064
GO:0030554	adenyl nucleotide binding	0.0045
GO:0032553	ribonucleotide binding	0.0047
GO:0032555	purine ribonucleotide binding	0.0047
GO:0032559	adenyl ribonucleotide binding	0.0033
GO:0050839	cell adhesion molecule binding	0.0409

This table displays significantly enriched GO terms (using a p-value<0.05) with respect to the “Molecular Function” GO category. (A) Molecular function of genes that show at least two-fold increase of expression value in the *green* compared to the *orange* sample. (B) Significantly enriched GO terms in the list of over-expressed genes that show a reduction of methylation. (C) Enriched terms found in the list of under-expressed genes (at least two fold) in the *green*. (D) Significant molecular function associated to the under-expressed genes that have an increase of methylation. No enrichment was found for GO categories that show both an increase of expression level and an increase of methylation and/or for under expressed genes that show a decrease of methylation.

## Discussion

The regulation of some physiological traits that we observed in aphids is controlled by environmental factors [30]. To this respect, the short-term maternal effect consists of environmental information being passed from the mother to the first and second generations of progenies (three telescopic generations are co-existing in aphids: the mother, the embryos and the nascent embryos inside the mature ones) [22], [23]. Our data argue in favor of a further level of gene regulation that consists in a long-term non-allelic heritability associated to extensive DNA methylation and orchestrated by the environmental pressures. The relatively fast selection of heritable variants, their reversal when the conditions change and the phenotypic memory re-enforce the epigenetic scenario without eliminating a parallel allele selection process. This suggests also that a massive transposition of mobile elements is not a valid scenario that might explain the *green* and the *orange* clonal phenotypes. The *green* variant is the result of a complex genetic network re-modeling, likely associated to site-specific methyl DNA assortment. Epigenetic marks heritable over a limited number of generations might constitute a solid mechanism for adaptation and fitness of aphids in a fluctuating environment. On the other hand, it has been suggested that methylation of cytosine in the gene body might affect the alternative use of promoters and the rate of polymerase that regulates alternative splicing (through recruitment of factors in the polymerase/spliceosome complex) [33]–[35]. The databases linked to this report will allow addressing these topics.

Moreover, the epigenetic regulation in the aphid model might include the endosymbiont component. Many cytosine methylases have been described in bacteria, phages and plasmids [36], [37]. The secondary endosymbionts are known to have a heavy load of transposases and shelter phages and plasmids [38], [39]. Phages are known to carry genes expressing methylases, like the multispecific cytosine methylase, functioning at sites like GGCC, GCGC or GGA/TGCCT/AC [37], [40]–[42] and the secondary endosymbiont *Candidatus hamiltonella* is known to harbor the methylase *Dcm* that modifies the second cytosine of CCA/TGG in the bacterial genome [36]. Many of these sites were found in the aphid genome pull down of methylated fragments. This collection enlarges the sequence specificities of DNA targets and raises the possibility that environmentally-induced bacteria/phage/plasmid methylases might modify the aphid host genome.

In conclusion, for each scaffold we have examined the location of methyl group densities present in promoters and/or in gene bodies and the variation of transcription in the vicinity of these methylations. Many methylated loci associated to enhancers likely regulate gene expression from a long distance in the linear sequence but act closely to the promoters by chromosome folding. These dynamic complex interactions make the correlation methylated DNA/gene expression very difficult to apprehend. Actual methodologies are still primitive to advance in this topic. However, we observed that methylation inversely correlates with gene expression for some analyzed metabolic pathways and seems to proceed in opposite ways for others. Moreover, if a correlation can be demonstrated in many cases, it seems absent for others. This suggests strongly that covalent modification of DNA induced by the environment might have a broad effect on genes by global modification of euchromatin/heterochromatin structure in chromosomes. However, this work allowed us to group the genes that vary between the two analyzed environments (22°C *versus* 8°C) in categories of molecular functions or biological processes. Specific metabolic pathways highlighted by GO analysis are consistent with environmental adaptability. We hypothesize that epigenetic stable marks might be transmitted through generations in clonality context and that the sexual barrier in fall could preserve those that are advantageous for the wave of clonal individuals the next spring. By this work, tools like the full differential transcriptome and the full methylome databases between environment-selected variants issued from a single founder mother might help to investigate the gene network re-organization in a fluctuating environment.

## Experimental Procedures

### Maintenance and propagation of aphids

Aphids *Acyrthosiphon pisum* were maintained on *Vicia faba* in cages in an incubation room at about 22°C +/−3°C, a light/dark photoperiodicity of 16/8 hours and 60% humidity. Aphids were raised at 8°C to select a predominant phenotype (green body color). See S1 Procedures for protocol details.

### Phenotypic selection, soluble extract injection and demography analysis

For the phenotype selection see S1 Procedures. For the injection experiments, 20 *green* adult aphids were roughly excised from the abdomen and the extracted material without the ovarioles was ground cautiously in a glass Potter in 200 µl of Ringer solution (115 mM NaCl, 3.5 mM KCl, 2.5 mM NaHCO_3_ and 4.5 mM CaCl_2_). After a brief centrifugation to eliminate membranes and debris, the supernatant was then injected in *orange* adult aphids using a micro-syringe under the microscope (magnification 10x) to deliver 10 to 20 µl. For experimental details, see S1 Procedures.

### Methyl-collector method for methyl DNA fragment enrichment and bisulfite sequencing

Total DNA was extracted from *A. pisum* and fragmented by *MseI* digestion, which leaves the CpG motif intact. The fragmented DNA was then affinity purified using the Methyl Collector kit (Active Motif), based on the recombinant protein containing a methyl CpG binding domain and a 6xHis tag. Following methyl CpG affinity precipitation, the resulting DNA fragments were linked with code bar adaptors in order to perform the pyrosequencing step (Beckman Coulter Genomics, Grenoble, France). Five individual preparations were pooled for each phenotype (*orange* and *green*). For detailed technical procedures, the bisulfite sequencing and also for the arguments that have guided the experimental choices see S1 Procedures.

### cDNA synthesis and Suppression Subtractive Hybridization (SSH)

Total RNA was extracted from *green* and *orange A. pisum* variants using the RNeasy Mini Kit (Qiagen), then the poly-adenylated mRNA was purified using the Oligotex mRNA mini-kit (Qiagen). cDNA synthesis and SSH were realized using the Clontech PCR-Select cDNA Subtraction Kit (Clontech) according to manufacturer‘s recommendations. Beckman Coulter Genomics (Grenoble, France) carried out the pyrosequencing and the analysis of the sequences. See S1 Procedures and Supplemental Data for the experimental details.

### Single stranded DNA library

The same cDNA synthesis protocol was used to create a single stranded (sst) DNA library from genomic or SSH products. According to the GS pyrosequencing protocol, DNA must first be transformed into a library of single-strand template DNA fragments (sstDNA) flanked with amplification and sequencing primer sequences. These sstDNA libraries were prepared using the GS Library Preparation kit (Roche Diagnostics GmbH) according to manufacturer’s recommendations. See the S1 Procedures for more details.

### emPCR and Sequencing run

Emulsion PCR (emPCR) corresponds to a clonal amplification of the sstDNA library. For sstDNA library sequencing application, the emPCR is carried out with the GS emPCR Kit I (Roche Diagnostics GmbH). The two Genomic samples or the two SSH samples were simultaneously sequenced in one region according to manufacturer’s recommendations. Two sets of bar codes adaptors (one for the *green* and the other for the *orange*) were used. The starting material was the accumulation of five independent experiments. See the S1 Procedures for more details.

### Methylome and transcriptome analysis

#### **Methylome**

The multiplex barcoded pyrosequencing was performed in a single run, with two samples tagged uniquely by multiplex identifiers (MID). The reads were directly mapped to the genome scaffolds of the *A. pisum* strain LSR1 version Acyr_1.0 with the program Razers [43]. By setting the default mismatch cutoff at 4%, about 95% of reads could be covered [44]. Dataset were deposited to NIH Short Read Archive under the references SRX719262 for methylated fragment found in the green variant and SRX719263 for methylated fragment found in the pink variant.

#### **Transcriptome**

The reads for both samples were assembled using the Newbler 2,0 program [45] after removal of the adapter sequences. The contigs were then searched for sequence similarity using BLAST [46] against the genome scaffolds of *A. pisum* strain LSR1 version Acyr_1.0. Contigs could be mapped unambiguously to the genome with default BLAST parameters and e-values ≤10^−50^ or better. To standardize the differential expression data between the two variants, we used DNA sequences that escaped the suppression step as internal control of the method (see detailed explanation in S1 Procedures). 864 contigs presenting high similarity (e- value < 1e-50) were filtered and retrieved by a reciprocal BLAST. The low number of similar reads between the two variants for each contig in this category was used to calibrate the size of the samples (see S1 Procedures). Dataset were deposited to NIH Short Read Archive under the references SRX719266 for the genes up-regulated in the pink variant and SRX719265 for the genes up-regulated in the green variant.

### Quantitative RT-PCR

RNA extraction was performed on 10 adult aphids using ISOLATE II RNA Mini Kit according to manufacturer’s instructions (BIOLINE). cDNA synthesis was done using the Tetro cDNA Synthesis Kit (BIOLINE). cDNA were amplified using SensiFAST SYBR No-ROX Kit (BIOLINE) and analyzed with Opticon Chromo 4 (Bio-Rad). The accumulation of each transcript was measured in three independent biological samples with three technical replicates. Expression of Ef1-1α, RPL7 and GAPDH was used to normalize the transcript level in each sample. Primer sequences are listed in table S4. Quantification and statistical analysis were calculated using the RqPCR Analysis Package [47].

## Gene Ontology enrichment

Methylome reads and transcriptome contigs were aligned to all known and predicted genes of *A. pisum*. A list of genes that show an increase of their expression value in the *orange* or the *green* sample (the level is indicated in the figures) has been selected for Gene Ontology term enrichment analysis. From this list of genes, two sub-lists were created: a list of genes that show a twofold increase of methylation and a list of genes that have a twofold decrease of methylation. Moreover, two extra lists of genes were also created: one for the genes that show an increase of expression value in the *orange* compared to the *green* sample and another one for the genes that show a decrease. The threshold cut off depends on the number of reads in contigs and is indicated in figures. From each of these lists, two sub-lists were created: one for the genes that show an increase of methylation and one for the genes that have a decrease of methylation.

A Gene Ontology term enrichment analysis has been performed on each of these lists. The Gene Ontology Enrichment Analysis Software Toolkit [48], [49], [50] was used to test the GO term enrichment within these given gene lists. Two ratios were compared in order to assess the probability of coherent changes: the ratio between the total number of genes found in the aphid genome by automatic annotation (N) and the number of these genes annotated by a given GO term (m) and, on the other side, the ratio between the number of genes in the list (n) and the number of these genes annotated by the GO term (k). The statistical method used to identify significantly enriched GO terms among the lists of genes is the hypergeometric test [48], [49], [50]. A p-value is provided to determine the degree of significance of the GO terms in the list.

## Supporting Information

S1 Fig **Bisulfite sequencing analysis.** The methylation was checked by bisulfite sequencing of few DNA fragments. Above, two fragments identified by our high through put method, have been analyzed. The match with the corresponding scaffold is shown. The quantitative analysis of the methylation was carried out with 20 individual bacterial clones for each original fragment and a relative percentage of methylation on each site is reported.
(PDF)

S2 Fig **Gbrowse examples showing transcriptomic and methylation data.** Representative genes decorates with the mapping of the exact matches corresponding to the methyl reads and the trascriptomic contigs. The reads were directly mapped to the genome scaffolds of *A. pisum* strain LSR1 version Acryr_2.0. Each pink and green triangles represent a red corresponding to the contigated fragments obtained after subtractive enrichment of transcript. Each pink and green traits represents a methyl read that is at least representing twice in pyrosequencing in the orange and the green.
(PDF)

S3 Fig **Gene Ontology term enrichment for genes showing a strong decrease in expression: hierarchical relationships in “Biological process”.** Selected genes are associated with at least 20 transcriptomic reads in one sample and no reads in the other sample. The graph represents GO term enrichment for the 88 genes for which expression decreases in the *green* compared to the *orange* aphids and displays their hierarchical relationships in “Biological process” GO category. Yellow boxes represent GO terms that are significantly enriched, with a p-value <0.1. The degree of color saturation of each node is positively correlated with the significance of enrichment of the corresponding GO term. Non-significant GO terms within the hierarchical tree are drawn as white boxes. Branches of the GO hierarchical tree without significant enriched GO terms are not shown. Edges stand for connections between different GO terms. Red edges stand for relationships between two enriched GO terms, black solid edges stand for relationships between enriched and non-enriched terms, black dashed edges stand for relationship between two un-enriched GO terms.
(PDF)

S4 Fig **Gene Ontology term enrichment for genes showing a strong decrease in expression: hierarchical relationships in “Molecular function”.** Same analysis than in S3 Fig. except the graph displays enriched GO terms and their hierarchical relationships in “Molecular function” GO category.
(PDF)

S5 Fig **Gene Ontology term enrichment for genes showing a strong increase in expression: hierarchical relationships in “Biological process”.** The terms of representation are described in the legend of S3 Fig. The graph represents the GO term enrichment for 21 genes with increased expression in the *green* compared the *orange* aphids and their hierarchical relationships in “Biological process” GO category.
(PDF)

S6 Fig **Gene Ontology term enrichment for genes showing a strong increase in expression: hierarchical relationships in “Molecular function”.** Same analysis than in S5 Fig. except the graph displays enriched GO terms and their hierarchical relationships in “Molecular function” GO category.
(PDF)

S7 Fig **Gene Ontology term enrichment for genes showing a slight to strong decrease of expression and their association to low methylation in gene body.** Gene Ontology term enrichment for genes showing a slight to strong decrease of gene expression (at least 5 transcriptomic reads in one sample and no reads in the other sample) in the *green* versus *orange* aphids associated to at least a twofold variation of methyl reads. Yellow boxes represent GO terms that are significantly enriched, with a p-value <0.1. The degree of color saturation of each node is positively correlated with the significance of enrichment of the corresponding GO term. Non-significant GO terms within the hierarchical tree are drawn as white boxes. Branches of the GO hierarchical tree without significant enriched GO terms are not shown. Edges stand for connections between different GO terms. Red edges stand for relationships between two enriched GO terms, black solid edges stand for relationships between enriched and non-enriched terms, black dashed edges stand for relationships between two un-enriched GO terms. The graph displays GO term enrichment for genes with a decreased expression in the *green* compared the *orange* aphids and a decrease of methyl reads in the gene body. This graph and the following ones (S8 to S14 figs.) refers to “Molecular Function” GO terms.
(PDF)

S8 Fig **Gene Ontology term enrichment for genes showing a slight to strong decrease of expression and their association to high methylation in gene body.** The “cut off” determinations for the analysis and the terms of the representation are described in the legend of S7 Fig.
(PDF)

S9 Fig **Gene Ontology term enrichment for genes showing a slight to strong decrease of expression and their association to low methylation in promoter.** The “cut off” determinations for the analysis and the terms of the representation are described in the legend of S7 Fig.
(PDF)

S10 Fig **Gene Ontology term enrichment for genes showing a slight to strong decrease of expression and their association to high methylation in promoter.** The “cut off” determinations for the analysis and the terms of the representation are described in the legend of S7 Fig.
(PDF)

S11 Fig **Gene Ontology term enrichment for genes showing a slight to strong increase of expression and their association to low methylation in gene body.** The “cut off” determinations for the analysis and the terms of the representation are described in the legend of S7 Fig.
(PDF)

S12 Fig **Gene Ontology term enrichment for genes showing a slight to strong increase of expression and their association to high methylation in gene body.** The “cut off” determinations for the analysis and the terms of the representation are described in the legend of S7 Fig.
(PDF)

S13 Fig **Gene Ontology term enrichment for genes showing a slight to strong increase of expression and their association to low methylation in promoter.** The “cut off” determinations for the analysis and the terms of the representation are described in the legend of S7 Fig.
(PDF)

S14 Fig **Gene Ontology term enrichment for genes showing a slight to strong increase of expression and their association to high methylation in promoter.** The “cut off” determinations for the analysis and the terms of the representation are described in the legend of S7 Fig.
(PDF)

S1 Table **List of methyl reads.** Lists of the retrieved methylated sequences found in the *green* and *orange* samples and the most represented in the genome. These reads match a large number of locations in the genome from 100 to 4500 times. This table is linked to the Fig. 2.
(DOCX)

S2 Table **List of methyl reads found at least twice in samples.** List of 21 000 methyl reads found at least identically twice in one or the other sample or in both. The sequences of each read is provided along with the number of copies in one or the other sample.
(XLS)

S3 Table **List of methyl reads found at least five times in samples.** List of 1200 methyl reads found at least identically five times in one or the other sample or in both. The sequences of each read is provided along with the number of copies in one or the other sample.
(XLS)

S4 Table **List of primers used for the q-PCR analysis.**
(DOCX)

S1 Procedures **Phenotypic selection, demography analysis and detailed molecular biology methods used in this report.**
(DOCX)

## References

[1] Bird A, Macleod D (2004). Reading the DNA methylation signal. Cold Spring Harb Symp Quant Biol 69, 113–118. 10.1101/sqb.2004.69.113 16117639

[2] Bastow R, Mylne JS, Lister C, Lippman Z, Martienssen RA, et al. (2004). Vernalization requires epigenetic silencing of FLC by histone methylation. Nature 427, 164–167. 10.1038/nature02269 14712277

[3] Becker C, Hagmann J, Muller J, Koenig D, Stegle O, et al. (2011). Spontaneous epigenetic variation in the Arabidopsis thaliana methylome. Nature 480, 245–249. 10.1038/nature10555 22057020

[4] Jacobsen SE, Sakai H, Finnegan EJ, Cao X, Meyerowitz EM (2000). Ectopic hypermethylation of flower-specific genes in Arabidopsis. Curr Biol 10, 179–186. 10.1016/s0960-9822(00)00324-9 10704409

[5] Johannes F, Porcher E, Teixeira FK, Saliba-Colombani V, Simon M, et al. (2009). Assessing the impact of transgenerational epigenetic variation on complex traits. PLoS Genet 5, e1000530. 10.1371/journal.pgen.1000530 PMC2696037 19557164

[6] Mathieu O, Reinders J, Caikovski M, Smathajitt C, Paszkowski J (2007). Transgenerational stability of the Arabidopsis epigenome is coordinated by CG methylation. Cell 130, 851–862. 10.1016/j.cell.2007.07.007 17803908

[7] Reinders J, Paszkowski J (2009). Unlocking the Arabidopsis epigenome. Epigenetics 4, 557–563. 10.4161/epi.4.8.10347 19934651

[8] Reinders J, Wulff BBH, Mirouze M, Marí-Ordóñez A, Dapp M (2009). Compromised stability of DNA methylation and transposon immobilization in mosaic Arabidopsis epigenomes. Genes Dev 23, 939–950. 10.1101/gad.524609 PMC2675864 19390088

[9] Shen H, He H, Li J, Chen W, Wang X, et al. (2012). Genome-wide analysis of DNA methylation and gene expression changes in two Arabidopsis ecotypes and their reciprocal hybrids. The Plant cell 24, 875–892. 10.1105/tpc.111.094870 PMC3336129 22438023

[10] Hunt BG, Brisson JA, Yi SV, Goodisman MA (2010). Functional conservation of DNA methylation in the pea aphid and the honeybee. Genome biology and evolution 2, 719–728. 10.1093/gbe/evq057 PMC2962555 20855427

[11] Kucharski R, Maleszka J, Foret S, Maleszka R (2008). Nutritional control of reproductive status in honeybees via DNA methylation. Science 319, 1827–1830. 10.1126/science.1153069 18339900

[12] Lyko F, Foret S, Kucharski R, Wolf S, Falckenhayn C, et al. (2010). The honey bee epigenomes: differential methylation of brain DNA in queens and workers. PLoS Biol 8, e1000506. 10.1371/journal.pbio.1000506 PMC2970541 21072239

[13] Lyko F, Maleszka R (2011) Insects as innovative models for functional studies of DNA methylation. Trends in Genetics 27:127–131. 21288591 10.1016/j.tig.2011.01.003

[14] Wang Y, Jorda M, Jones PL, Maleszka R, Ling X, et al. (2006). Functional CpG methylation system in a social insect. Science 314, 645–647. 10.1126/science.1135213 17068262

[15] Fazzari MJ, Greally JM (2004). Epigenomics: beyond CpG islands. Nature reviews Genetics 5, 446–455. 10.1038/nrg1349 15153997

[16] Jaenisch R, Bird A (2003). Epigenetic regulation of gene expression: how the genome integrates intrinsic and environmental signals. Nature genetics 33 Suppl, 245–254. 10.1038/ng1089 12610534

[17] Rando OJ, Verstrepen KJ (2007). Timescales of genetic and epigenetic inheritance. Cell 128, 655–668. 10.1016/j.cell.2007.01.023 17320504

[18] Weaver ICG, Cervoni N, Champagne FA, D’Alessio AC, Sharma S, et al. (2004). Epigenetic programming by maternal behavior. Nat Neurosci 7, 847–854. 10.1038/nn1276 15220929

[19] Morgan HD, Sutherland HG, Martin DI, Whitelaw E (1999). Epigenetic inheritance at the agouti locus in the mouse. Nature genetics 23, 314–318. 10.1038/15490 10545949

[20] Richards EJ (2006). Inherited epigenetic variation–revisiting soft inheritance. Nat Rev Genet 7, 395–401. 10.1038/nrg1834 16534512

[21] Vilkaitis G, Suetake I, Klimasauskas S, Tajima S (2005). Processive methylation of hemimethylated CpG sites by mouse Dnmt1 DNA methyltransferase. J Biol Chem 280, 64–72. 10.1074/jbc.M411126200 15509558

[22] Dixon AFG (1973). Biology of aphids. In Studies in Biology, E. Arnold, ed. (London).

[23] Dixon AFG (1998). Aphid Ecology (London: Chapman & Hall).

[24] Miura T, Braendle C, Shingleton A, Sisk G, Kambhampati S., et al. (2003). A comparison of parthenogenetic and sexual embryogenesis of the pea aphid Acyrthosiphon pisum (Hemiptera: Aphidoidea). J Exp Zool B Mol Dev Evol 295, 59–81. 10.1002/jez.b.3 12548543

[25] Braendle C, Miura T, Bickel R, Shingleton AW, Kambhampati S, et al. (2003). Developmental origin and evolution of bacteriocytes in the aphid-Buchnera symbiosis. PLoS Biol 1, E21. 10.1371/journal.pbio.0000021 PMC212699 14551917

[26] Dale C, Moran NA (2006). Molecular interactions between bacterial symbionts and their hosts. Cell 126, 453–465. 10.1016/j.cell.2006.07.014 16901780

[27] Moran N, Wernegreen J (2000). Lifestyle evolution in symbiotic bacteria: insights from genomics. Trends Ecol Evol (Amst) 15, 321–326. 10.1016/s0169-5347(00)01902-9 10884696

[28] Scarborough CL, Ferrari J, Godfray HCJ (2005). Aphid protected from pathogen by endosymbiont. Science 310, 1781. 10.1126/science.1120180 16357252

[29] Tsuchida T, Koga R, Horikawa M, Tsunoda T, Maoka T, et al. (2010). Symbiotic bacterium modifies aphid body color. Science 330, 1102–1104. 10.1126/science.1195463 21097935

[30] Dombrovsky A, Arthaud L, Ledger TN, Tares S, Robichon A (2009). Profiling the repertoire of phenotypes influenced by environmental cues that occur during asexual reproduction. Genome Res 19, 2052–2063. 10.1101/gr.091611.109 PMC2775594 19635846

[31] Moran NA, Jarvik T (2010). Lateral transfer of genes from fungi underlies carotenoid production in aphids. Science 328, 624–627. 10.1126/science.1187113 20431015

[32] Valmalette JC, Dombrovsky A, Brat P, Mertz C, Capovilla M, et al. (2012). Light- induced electron transfer and ATP synthesis in a carotene synthesizing insect. Scientific report 2012. 10.1038/srep00579 PMC3420219 22900140

[33] Kornblihtt AR (2005). Promoter usage and alternative splicing. Curr Opin Cell Biol 17, 262–268. 10.1016/j.ceb.2005.04.014 15901495

[34] Maunakea AK, Nagarajan RP, Bilenky M, Ballinger TJ, D’Souza C, et al. (2010). Conserved role of intragenic DNA methylation in regulating alternative promoters. Nature 466, 253–257. 10.1038/nature09165 PMC3998662 20613842

[35] Perales R, Bentley D (2009). “Cotranscriptionality”: the transcription elongation complex as a nexus for nuclear transactions. Mol Cell 36, 178–191. 10.1016/j.molcel.2009.09.018 PMC2770090 19854129

[36] Bhagwat AS, Sohail A, Roberts RJ (1986). Cloning and characterization of the dcm locus of Escherichia coli K-12. J Bacteriol 166, 751–755. 10.1128/jb.166.3.751-755.1986 PMC215190 3011742

[37] Cohen HM, Tawfik DS, Griffiths AD (2002). Promiscuous methylation of non-canonical DNA sites by HaeIII methyltransferase. Nucleic Acids Res 30, 3880–3885. 10.1093/nar/gkf507 PMC137429 12202773

[38] Degnan PH, Moran NA (2008). Diverse phage-encoded toxins in a protective insect endosymbiont. Appl Environ Microbiol 74, 6782–6791. 10.1128/AEM.01285-08 PMC2576707 18791000

[39] Oliver KM, Degnan PH, Burke GR, Moran NA (2010). Facultative symbionts in aphids and the horizontal transfer of ecologically important traits. Annu Rev Entomol 55, 247–266. 10.1146/annurev-ento-112408-085305 19728837

[40] Lange C, Noyer-Weidner M, Trautner TA, Weiner M, Zahler SA (1991). M.H2I, a multispecific 5C–DNA methyltransferase encoded by Bacillus amyloliquefaciens phage H2. Gene 100, 213–218. 10.1016/0378-1119(91)90369-m 2055471

[41] Schumann J, Walter J, Willert J, Wild C, Koch D, et al. (1996). M.BssHII, a multispecific cytosine-C5-DNA-methyltransferase with unusual target recognizing properties. J Mol Biol 257, 949–959. 10.1006/jmbi.1996.0214 8632477

[42] Terschüren PA, Noyer-Weidner M, Trautner TA (1987). Recombinant derivatives of Bacillus subtilis phage Z containing the DNA methyltransferase genes of related methylation-proficient phages. J Gen Microbiol 133, 945–952. 10.1099/00221287-133-4-945 3116167

[43] Weese D, Emde AK, Rausch T, Döring A, Reinert K (2009). RazerS–fast read mapping with sensitivity control. Genome Res 19, 1646–1654. 10.1101/gr.088823.108 PMC2752123 19592482

[44] Niu B, Fu L, Sun S, Li W (2010). Artificial and natural duplicates in pyrosequencing reads of metagenomic data. BMC Bioinformatics 11, 187. 10.1186/1471-2105-11-187 PMC2874554 20388221

[45] Margulies M, Egholm M, Altman WE, Attiya S, Bader JS, et al. (2005). Genome sequencing in microfabricated high-density picolitre reactors. Nature 437, 376–380. 10.1038/nature03959 PMC1464427 16056220

[46] Altschul SF, Gish W, Miller W, Myers EW, Lipman DJ (1990). Basic local alignment search tool. J Mol Biol 215, 403–410. 10.1016/S0022-2836(05)80360-2 2231712

[47] Hilliou F, Tran T (2013). RqPCRAnalysis: Analysis of Quantitative Real-time PCR Data. Paper Proceedings of International Conference on Bioinformatics Models, Methods and Algorithms, BIOSTEC 2013, ISBN 978–989–8565–35–8. DOI:doi:10.5220/0004312002020211..

[48] Zheng Q, Wang XJ (2008). GOEAST: a web-based software toolkit for Gene Ontology enrichment analysis. Nucleic Acids Res 36, W358–363. 10.1093/nar/gkn276 PMC2447756 18487275

[49] Ashburner M, Ball CA, Blake JA, Botstein D, Butler H, et al. (2000). Gene ontology: tool for the unification of biology. The Gene Ontology Consortium. Nature genetics 25, 25–29. 10.1038/75556 PMC3037419 10802651

[50] Consortium IAG (2010). Genome sequence of the pea aphid Acyrthosiphon pisum. PLoS Biol 8, e1000313. 10.1371/journal.pbio.1000313 PMC2826372 20186266

