# Supplementary figures and images for: Environmentally Selected Aphid Variants in Clonality Context Display Differential Patterns of Methylation in the Genome
Source: PLoS One. 2014 Dec 31;9(12):e115022. doi: 10.1371/journal.pone.0115022 (PMC4281257; doi:10.1371/journal.pone.0115022)

Figure S1

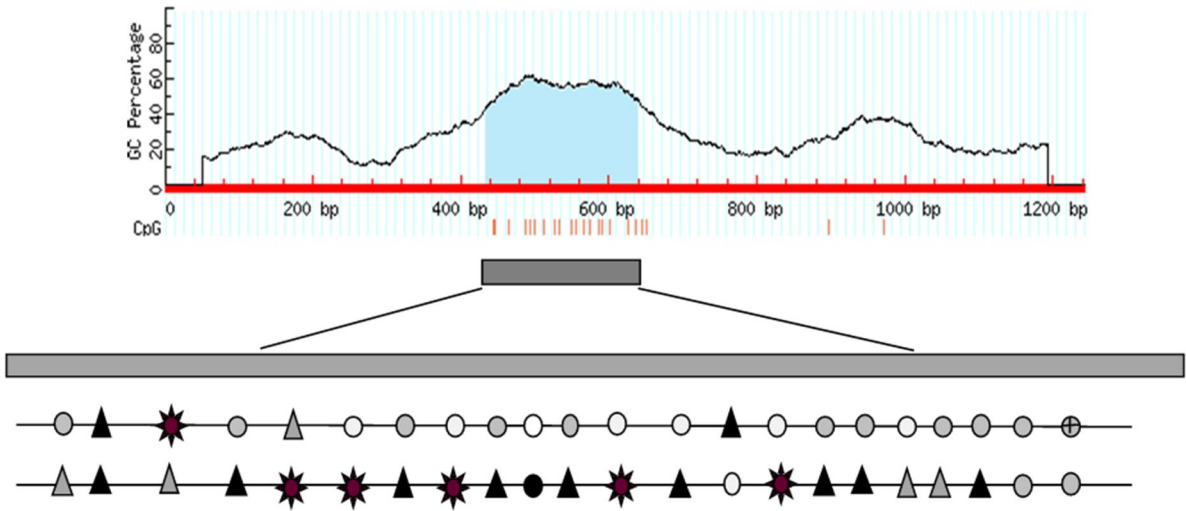

above: *orange*; below: *green*

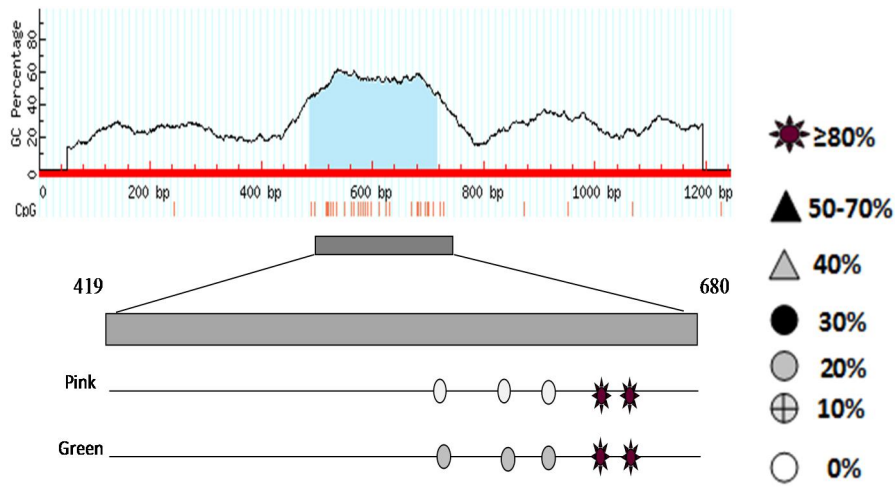

Supplement: S1 Fig — Bisulfite sequencing analysis. The methylation was checked by bisulfite sequencing of few DNA fragments. Above, two fragments identified by our high through put method, have been analyzed. The match with the corresponding scaffold is shown. The quantitative analysis of the methylation was carried out with 20 individual bacterial clones for each original fragment and a relative percentage of methylation on each site is reported. (PDF) [file pone.0115022.s001.pdf]

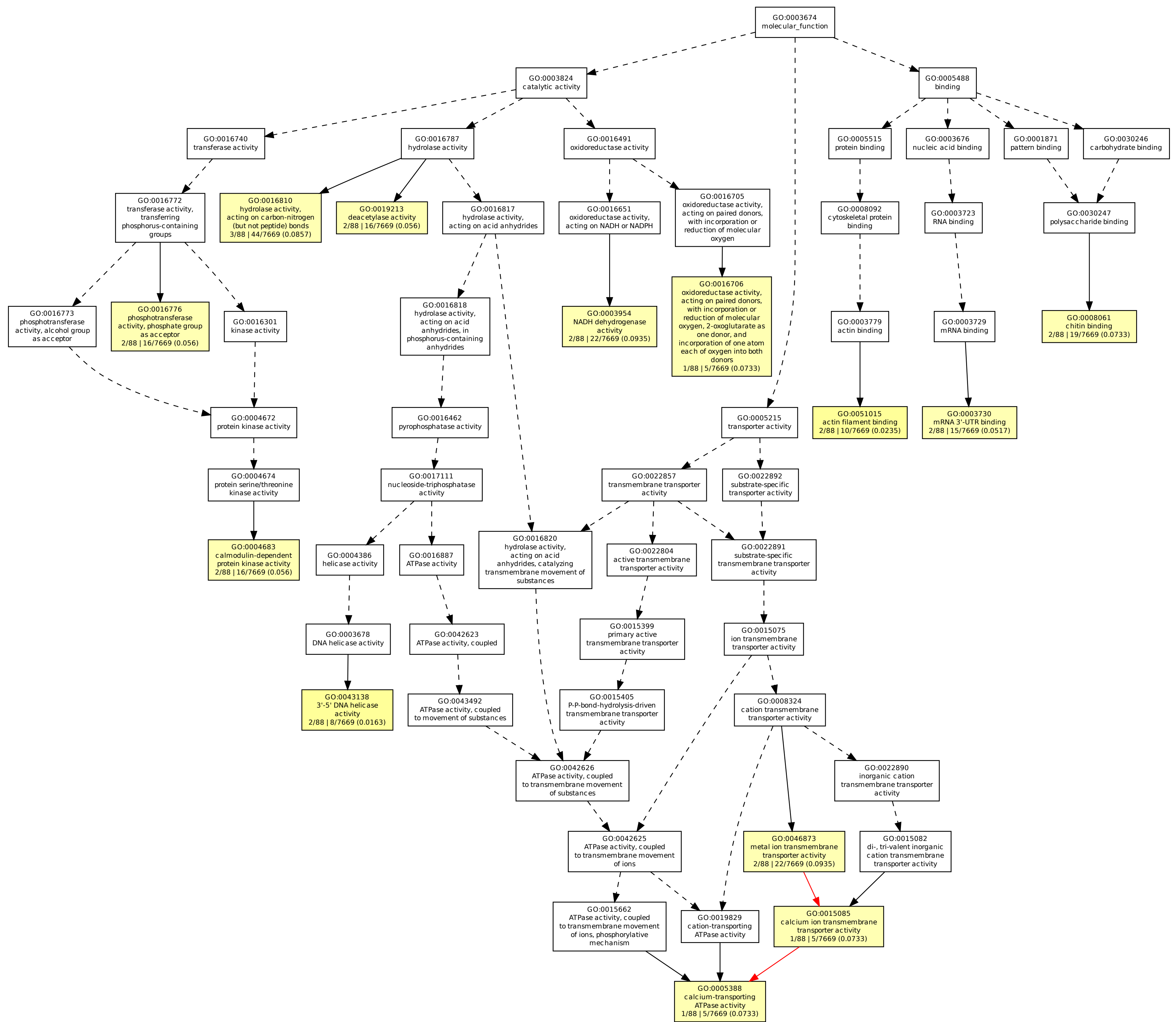

Supplement: S4 Fig — Gene Ontology term enrichment for genes showing a strong decrease in expression: hierarchical relationships in “Molecular function”. Same analysis than in S3 Fig. except the graph displays enriched GO terms and their hierarchical relationships in “Molecular function” GO category. (PDF) [file pone.0115022.s004.pdf]

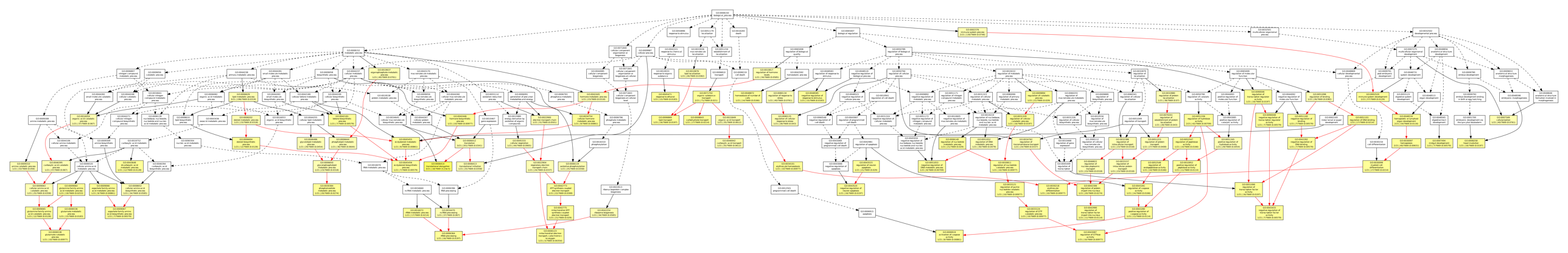

Supplement: S5 Fig — Gene Ontology term enrichment for genes showing a strong increase in expression: hierarchical relationships in “Biological process”. The terms of representation are described in the legend of S3 Fig. The graph represents the GO term enrichment for 21 genes with increased expression in the green compared the orange aphids and their hierarchical relationships in “Biological process” GO category. (PDF) [file pone.0115022.s005.pdf]

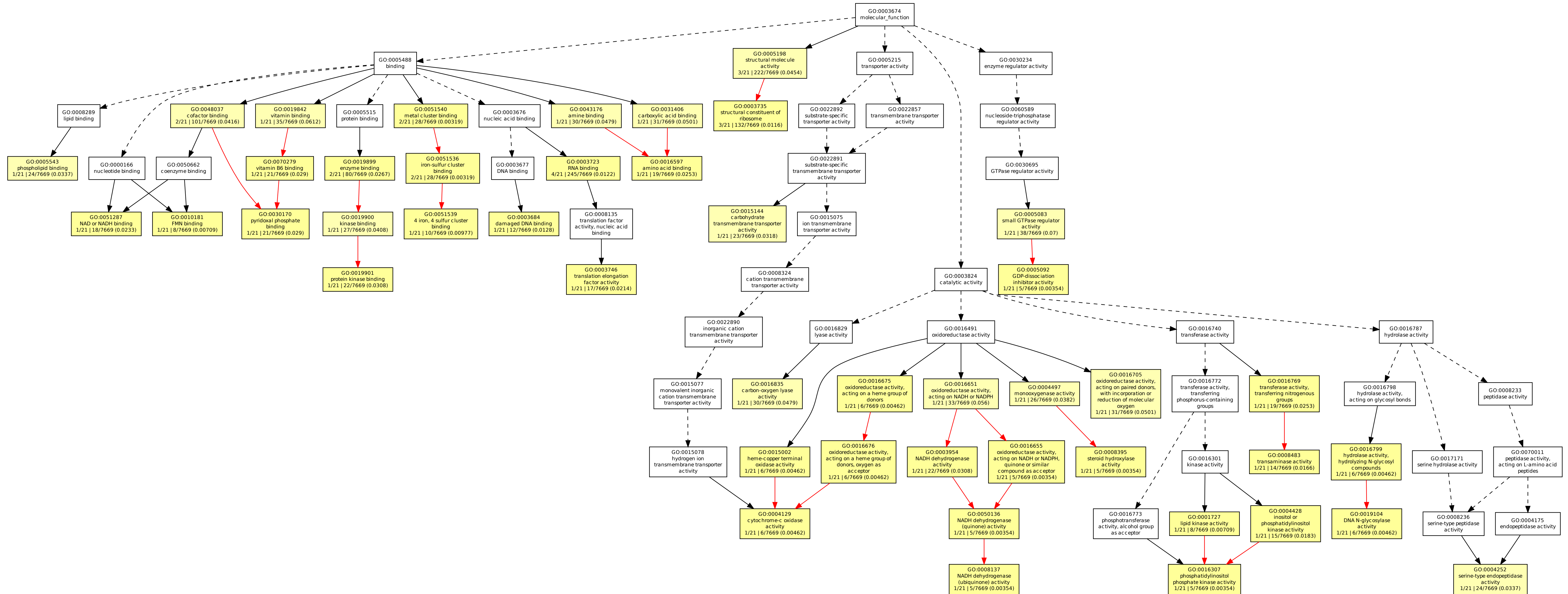

Supplement: S6 Fig — Gene Ontology term enrichment for genes showing a strong increase in expression: hierarchical relationships in “Molecular function”. Same analysis than in S5 Fig. except the graph displays enriched GO terms and their hierarchical relationships in “Molecular function” GO category. (PDF) [file pone.0115022.s006.pdf]

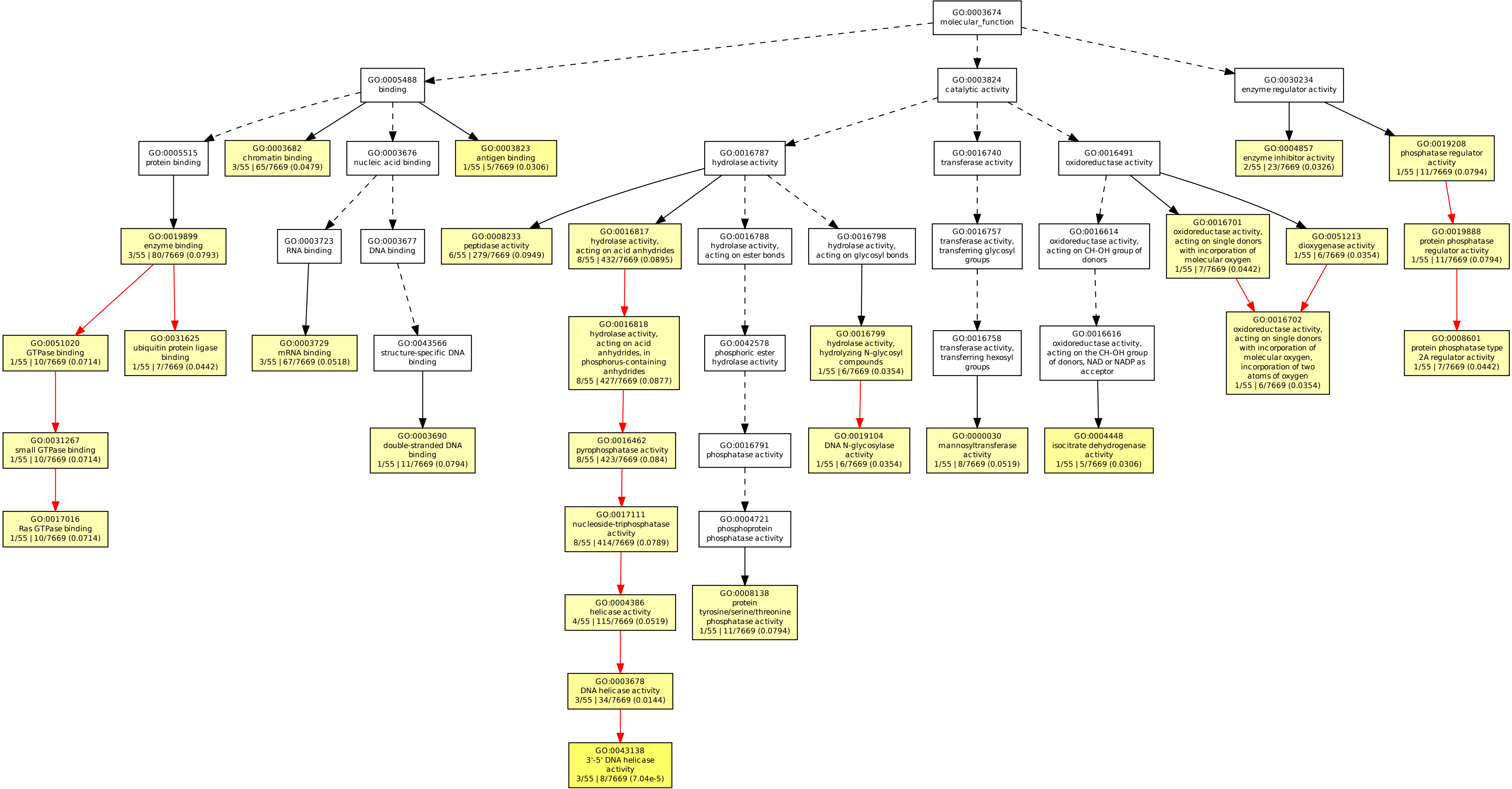

Supplement: S7 Fig — Gene Ontology term enrichment for genes showing a slight to strong decrease of expression and their association to low methylation in gene body. Gene Ontology term enrichment for genes showing a slight to strong decrease of gene expression (at least 5 transcriptomic reads in one sample and no reads in the other sample) in the green versus orange aphids associated to at least a twofold variation of methyl reads. Yellow boxes represent GO terms that are significantly enriched, with a p-value <0.1. The degree of color saturation of each node is positively correlated with the significance of enrichment of the corresponding GO term. Non-significant GO terms within the hierarchical tree are drawn as white boxes. Branches of the GO hierarchical tree without significant enriched GO terms are not shown. Edges stand for connections between different GO terms. Red edges stand for relationships between two enriched GO terms, black solid edges stand for relationships between enriched and non-enriched terms, black dashed edges stand for relationships between two un-enriched GO terms. The graph displays GO term enrichment for genes with a decreased expression in the green compared the orange aphids and a decrease of methyl reads in the gene body. This graph and the following ones (S8 to S14 figs.) refers to “Molecular Function” GO terms. (PDF) [file pone.0115022.s007.pdf]

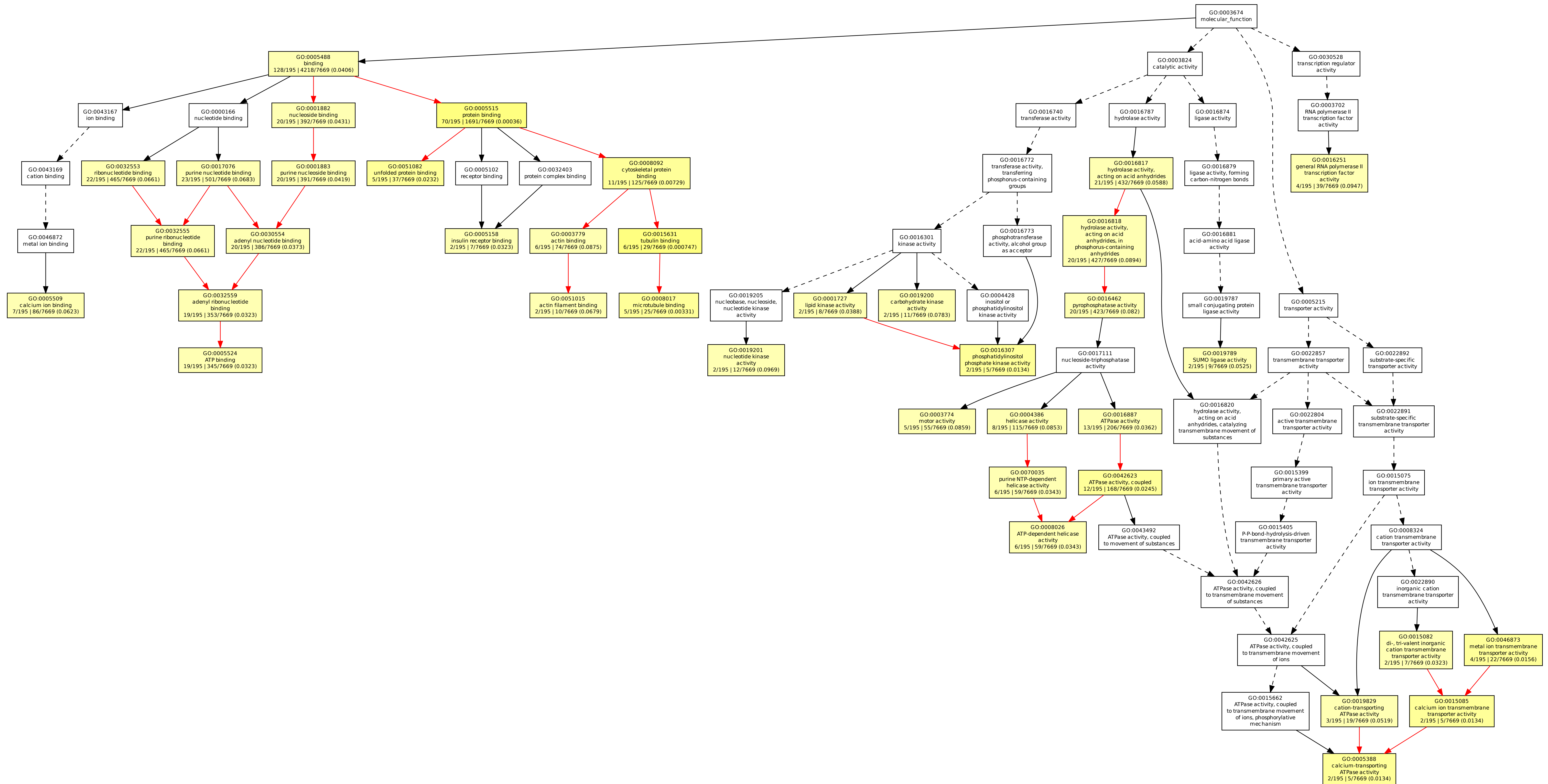

Supplement: S8 Fig — Gene Ontology term enrichment for genes showing a slight to strong decrease of expression and their association to high methylation in gene body. The “cut off” determinations for the analysis and the terms of the representation are described in the legend of S7 Fig. (PDF) [file pone.0115022.s008.pdf]

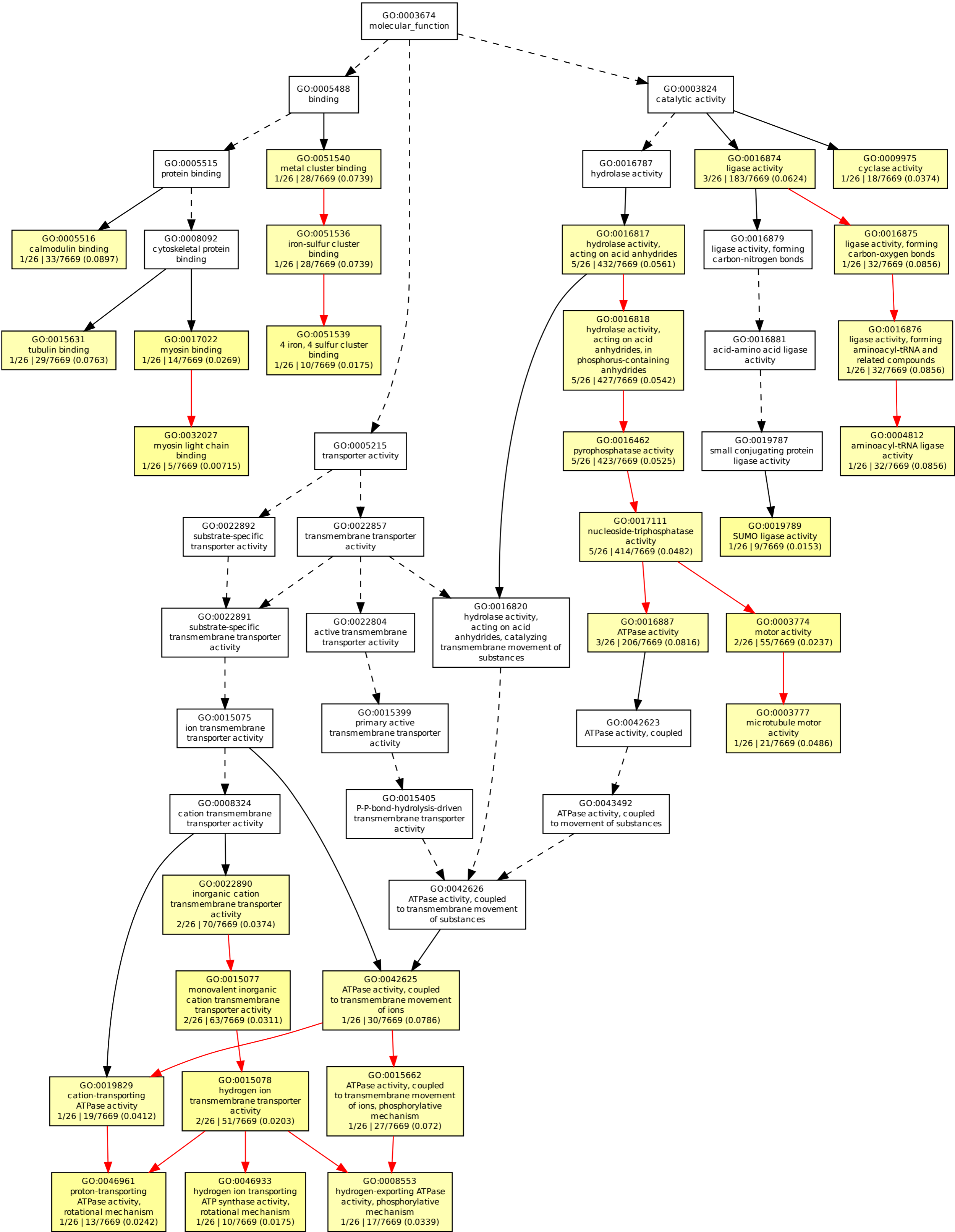

Supplement: S9 Fig — Gene Ontology term enrichment for genes showing a slight to strong decrease of expression and their association to low methylation in promoter. The “cut off” determinations for the analysis and the terms of the representation are described in the legend of S7 Fig. (PDF) [file pone.0115022.s009.pdf]

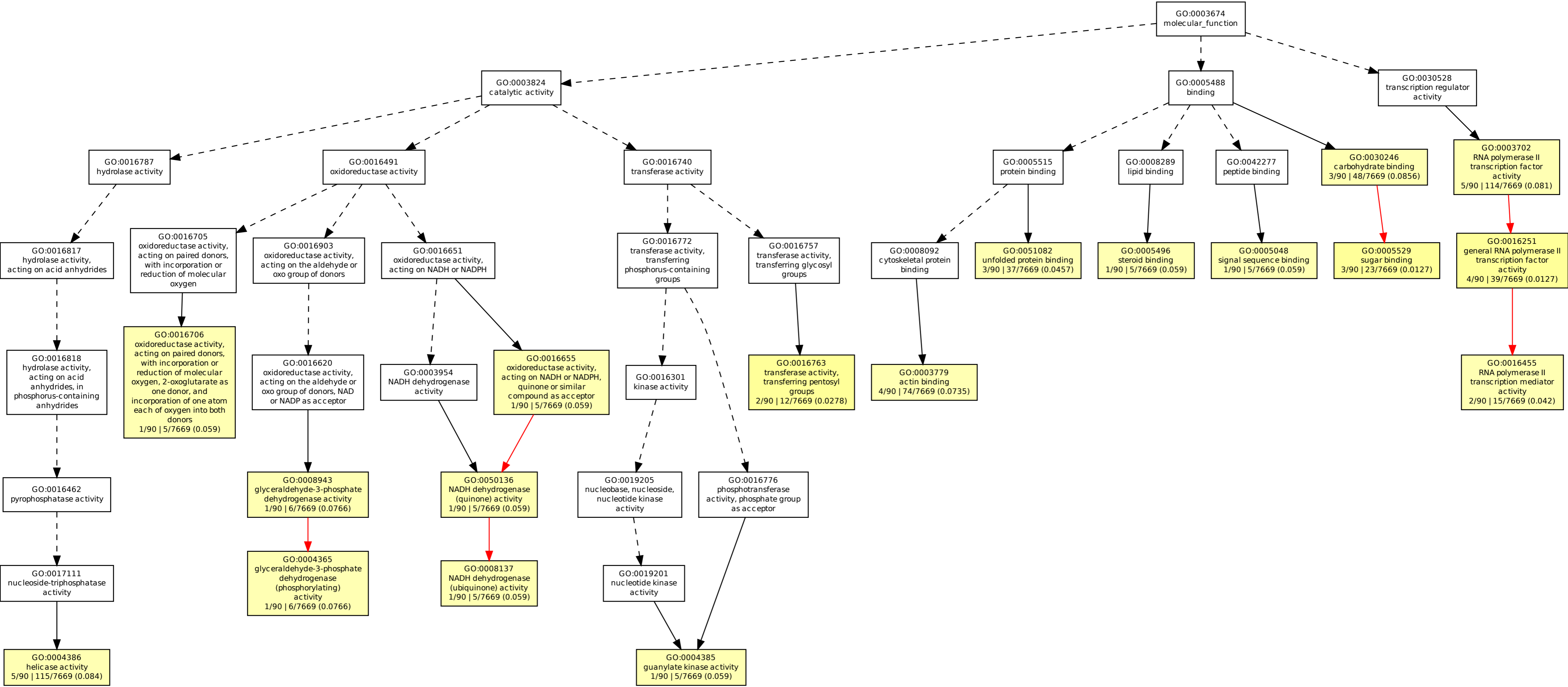

Supplement: S10 Fig — Gene Ontology term enrichment for genes showing a slight to strong decrease of expression and their association to high methylation in promoter. The “cut off” determinations for the analysis and the terms of the representation are described in the legend of S7 Fig. (PDF) [file pone.0115022.s010.pdf]

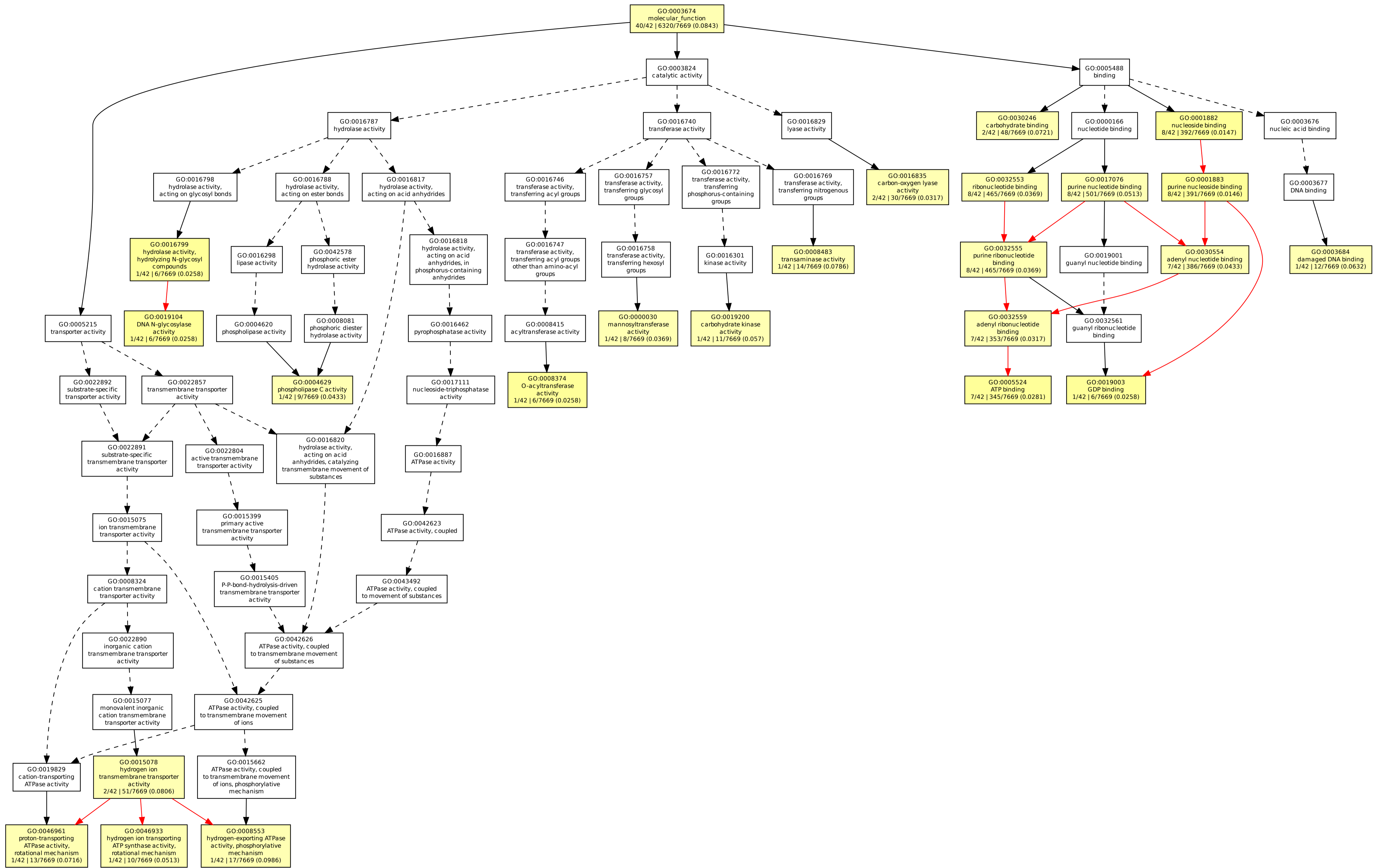

Supplement: S11 Fig — Gene Ontology term enrichment for genes showing a slight to strong increase of expression and their association to low methylation in gene body. The “cut off” determinations for the analysis and the terms of the representation are described in the legend of S7 Fig. (PDF) [file pone.0115022.s011.pdf]

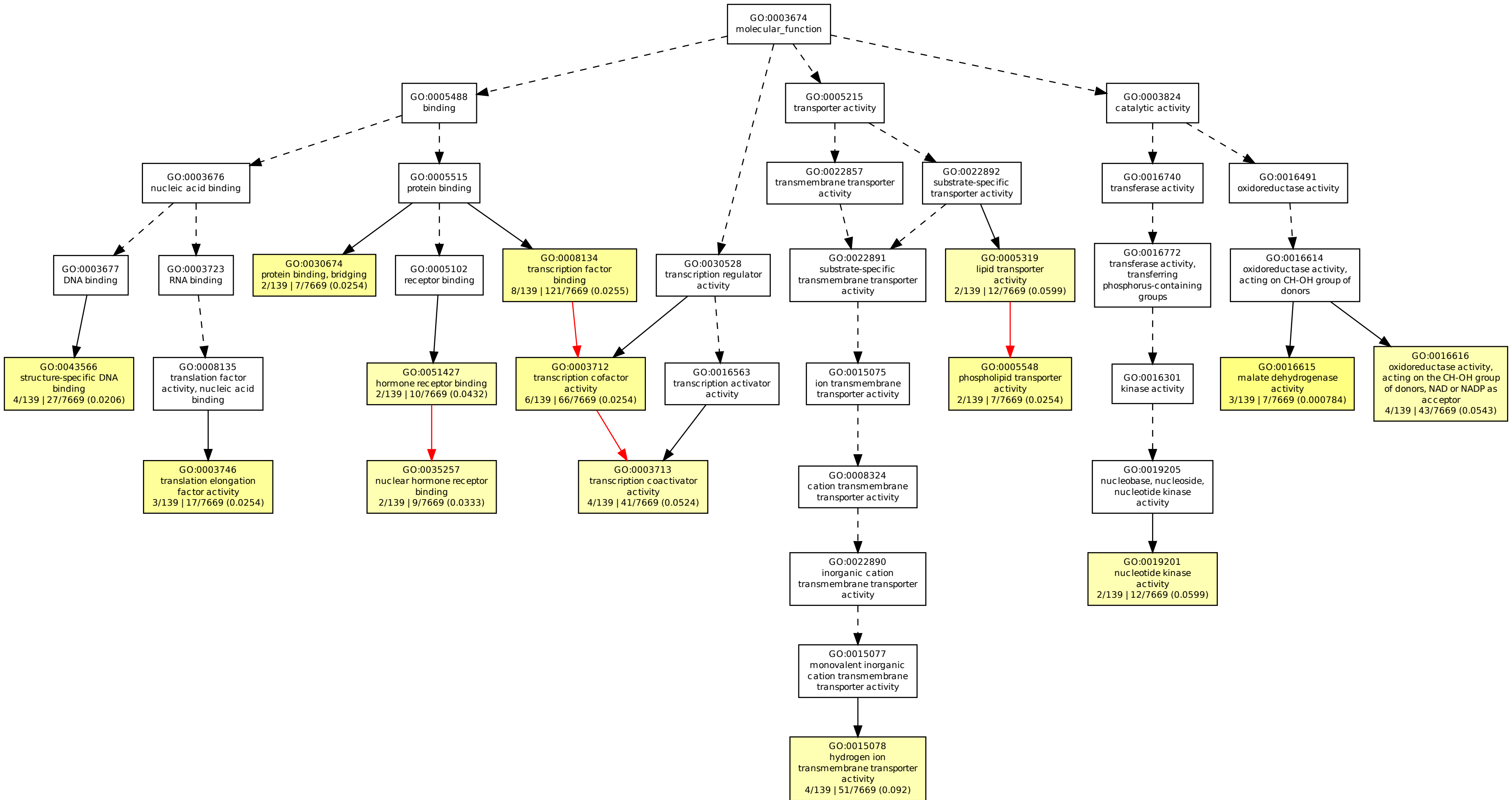

Supplement: S12 Fig — Gene Ontology term enrichment for genes showing a slight to strong increase of expression and their association to high methylation in gene body. The “cut off” determinations for the analysis and the terms of the representation are described in the legend of S7 Fig. (PDF) [file pone.0115022.s012.pdf]

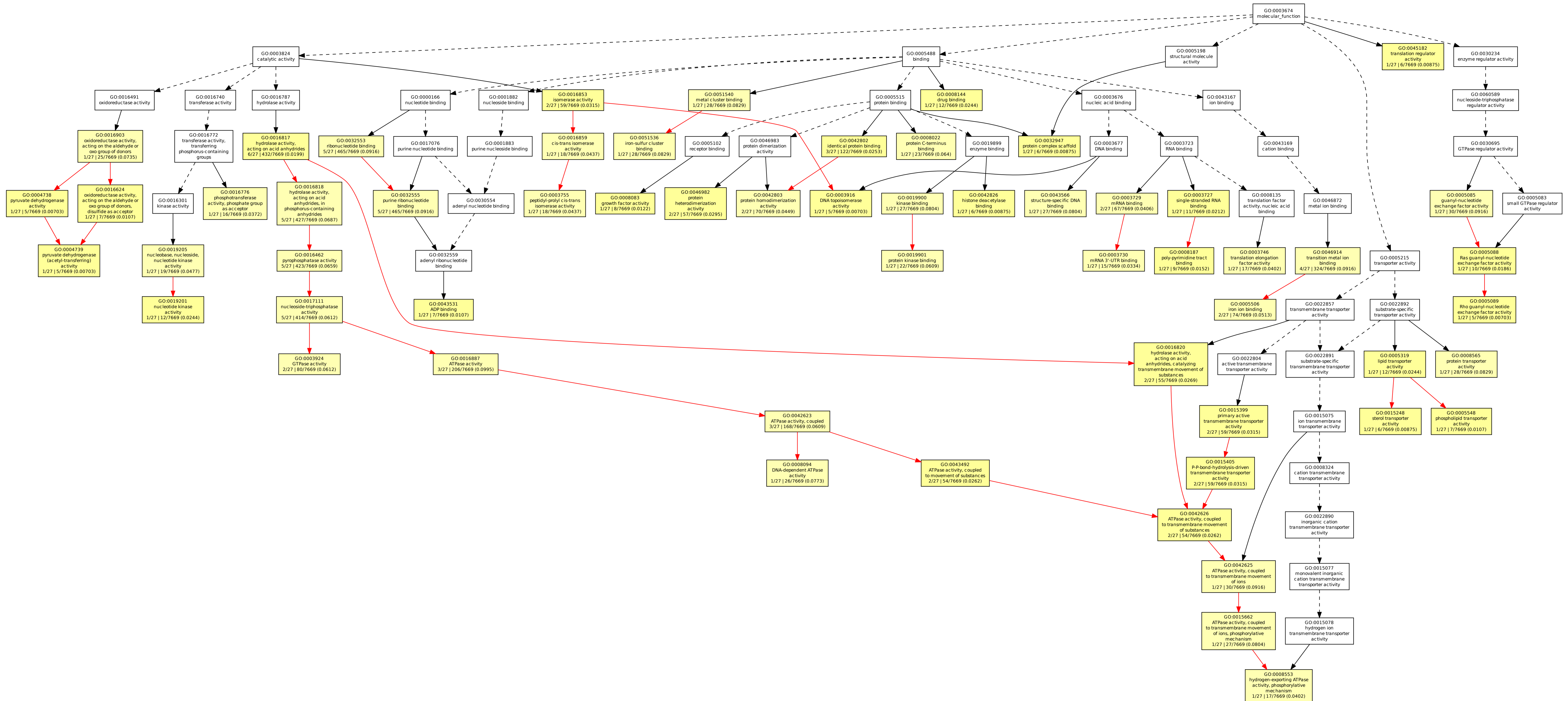

Supplement: S13 Fig — Gene Ontology term enrichment for genes showing a slight to strong increase of expression and their association to low methylation in promoter. The “cut off” determinations for the analysis and the terms of the representation are described in the legend of S7 Fig. (PDF) [file pone.0115022.s013.pdf]

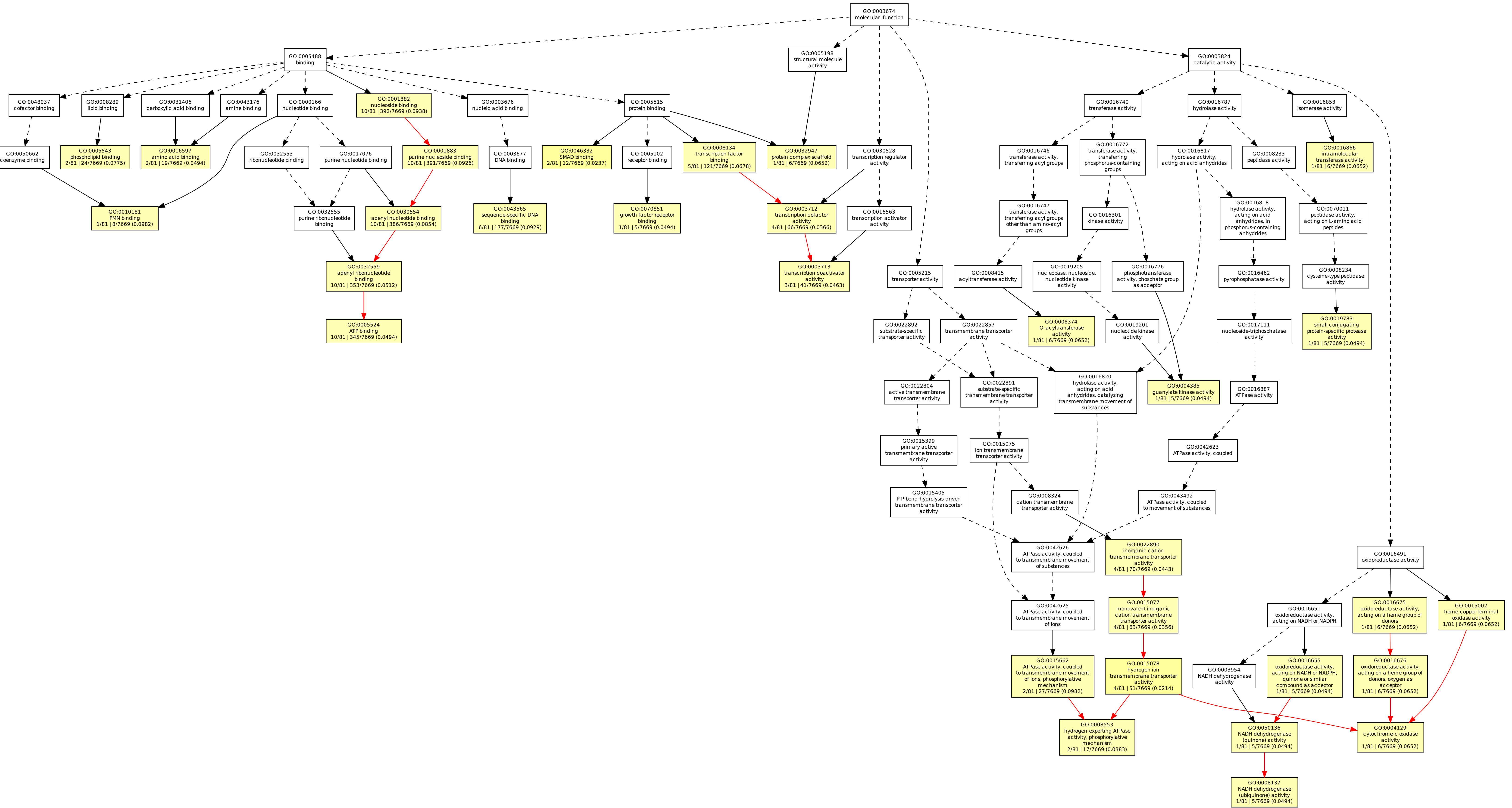

Supplement: S14 Fig — Gene Ontology term enrichment for genes showing a slight to strong increase of expression and their association to high methylation in promoter. The “cut off” determinations for the analysis and the terms of the representation are described in the legend of S7 Fig. (PDF) [file pone.0115022.s014.pdf]

**
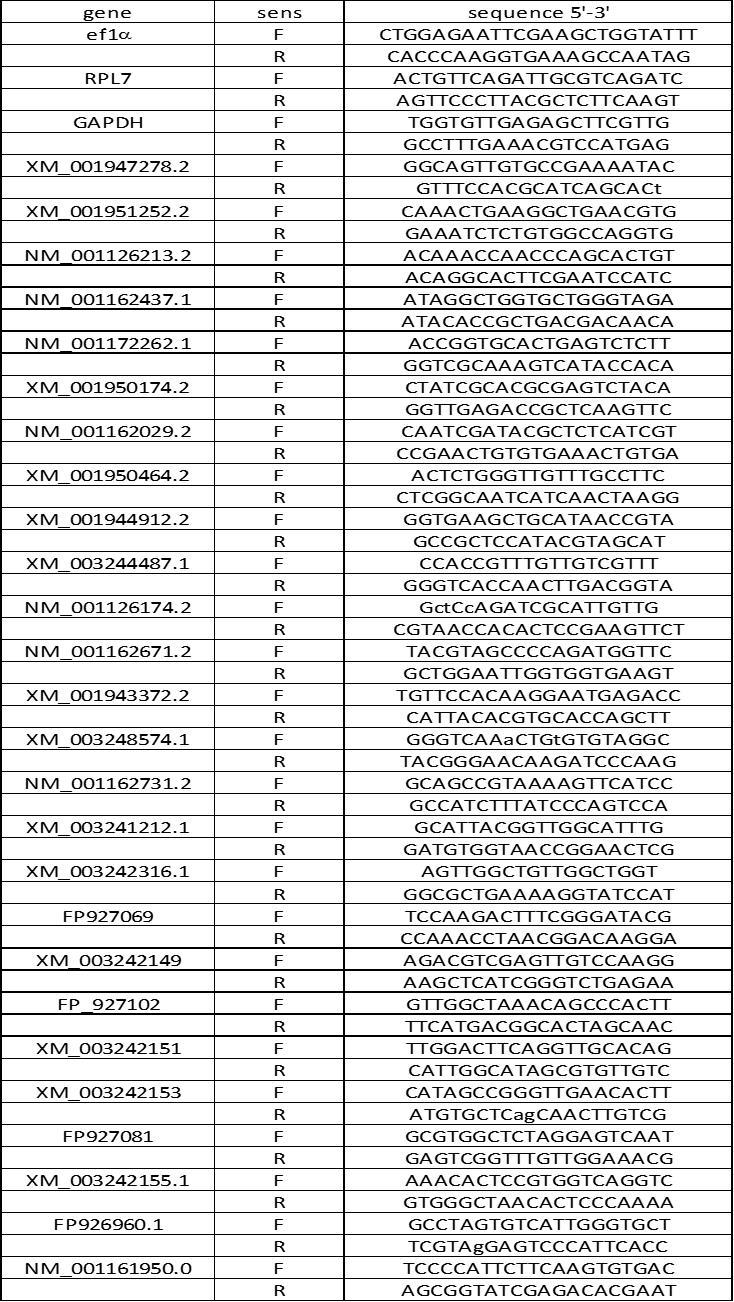
Table S4. List of primers used for the q-PCR analysis**

Supplement: S4 Table — List of primers used for the q-PCR analysis. (DOCX) [file pone.0115022.s018.docx]
