# Supplementary material for: Environmentally Selected Aphid Variants in Clonality Context Display Differential Patterns of Methylation in the Genome
Source: PLoS One. 2014 Dec 31;9(12):e115022. doi: 10.1371/journal.pone.0115022 (PMC4281257; doi:10.1371/journal.pone.0115022)

a. Gbrowse extract : over methylation in green and up regulation in orange

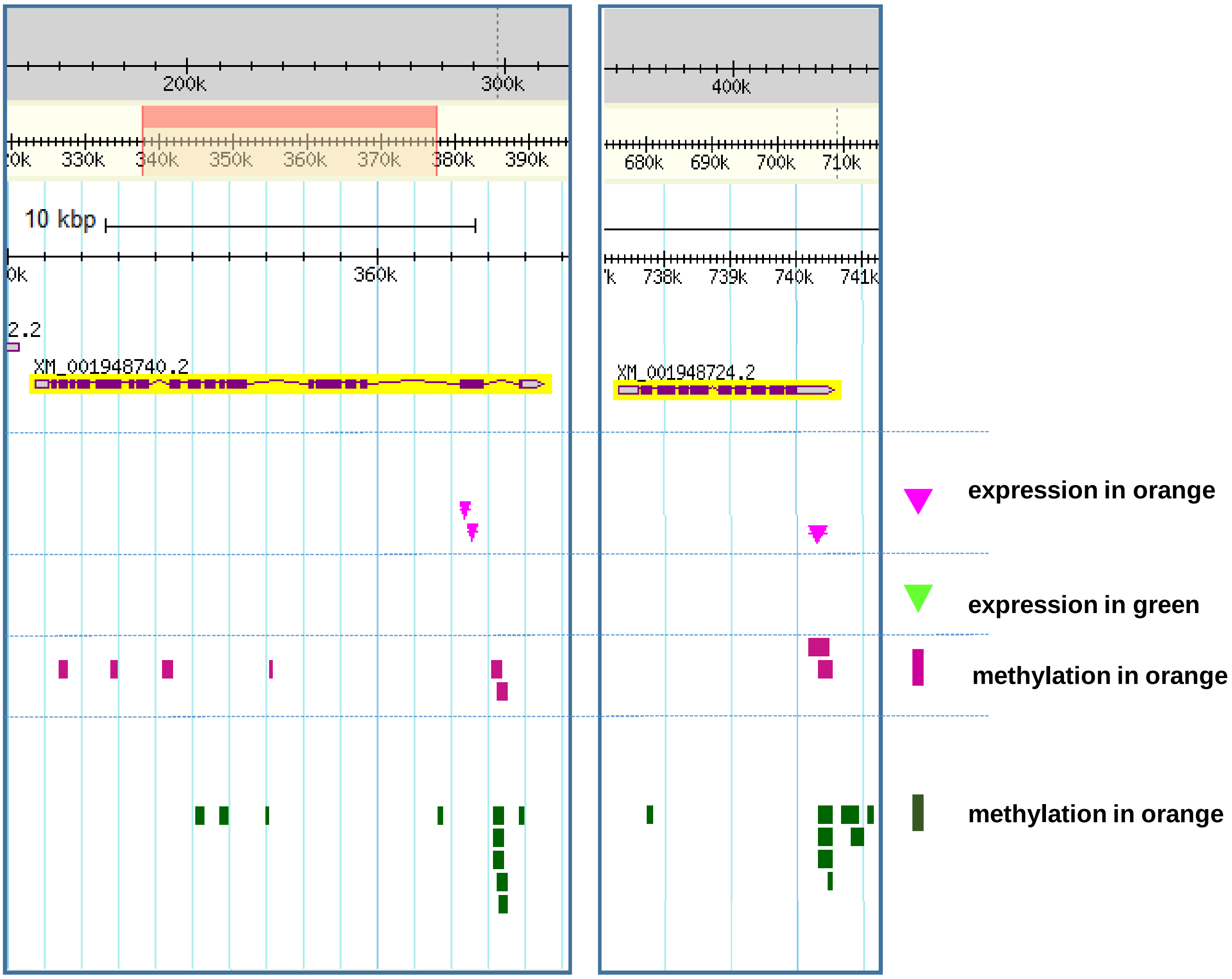

b. Gbrowse extract : difference in methylation and non difference in expression

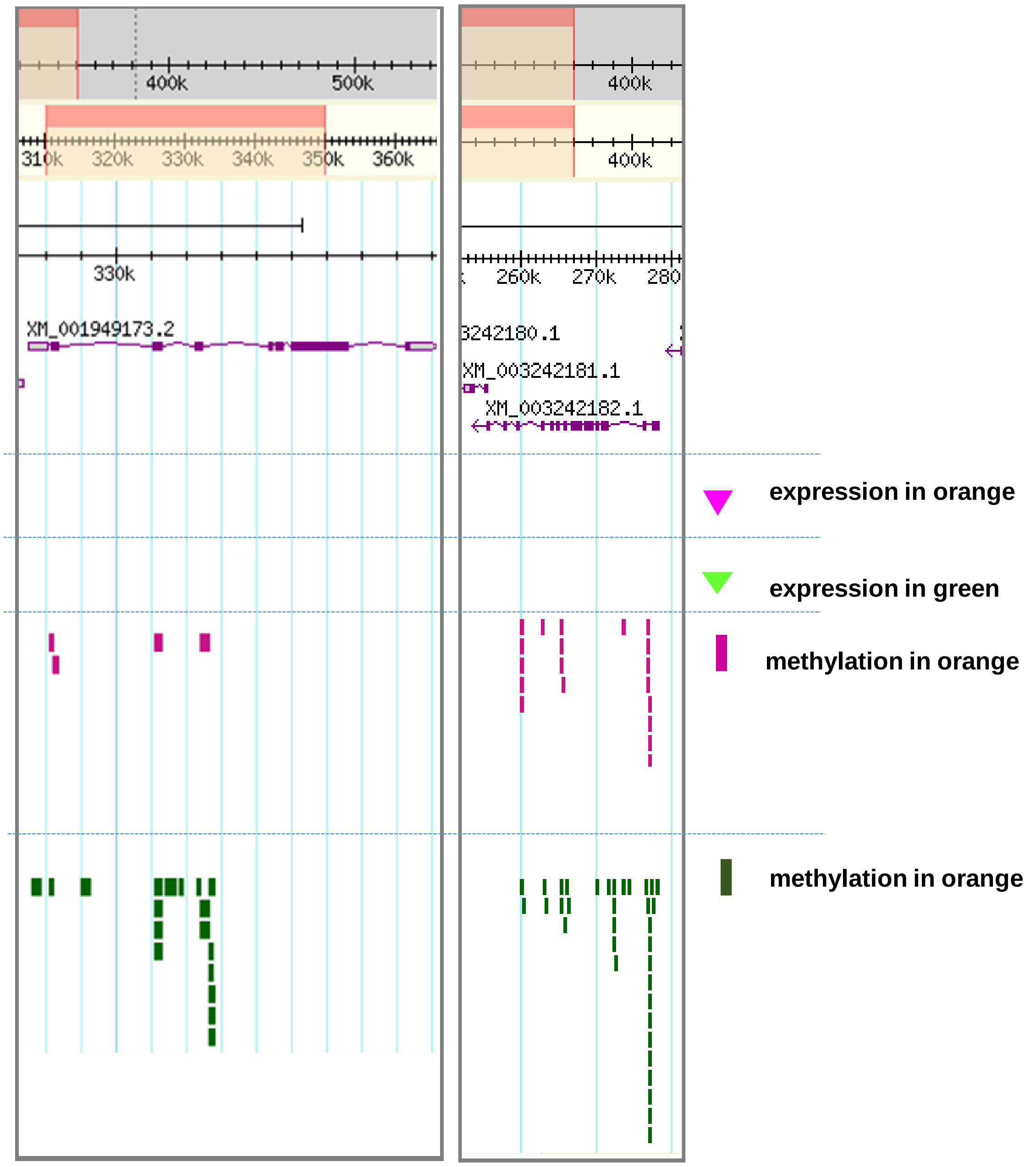

c. Gbrowse extract : no difference in methylation and difference in expression

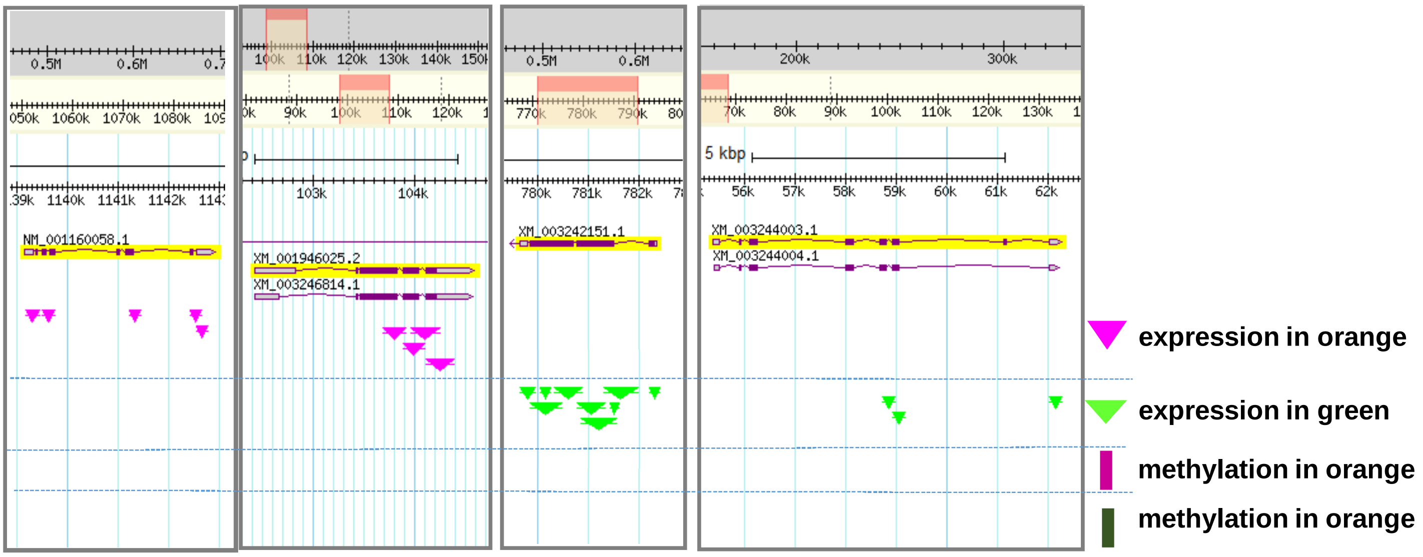

d. Gbowse ectract : overmethylation in green and overexpression in green

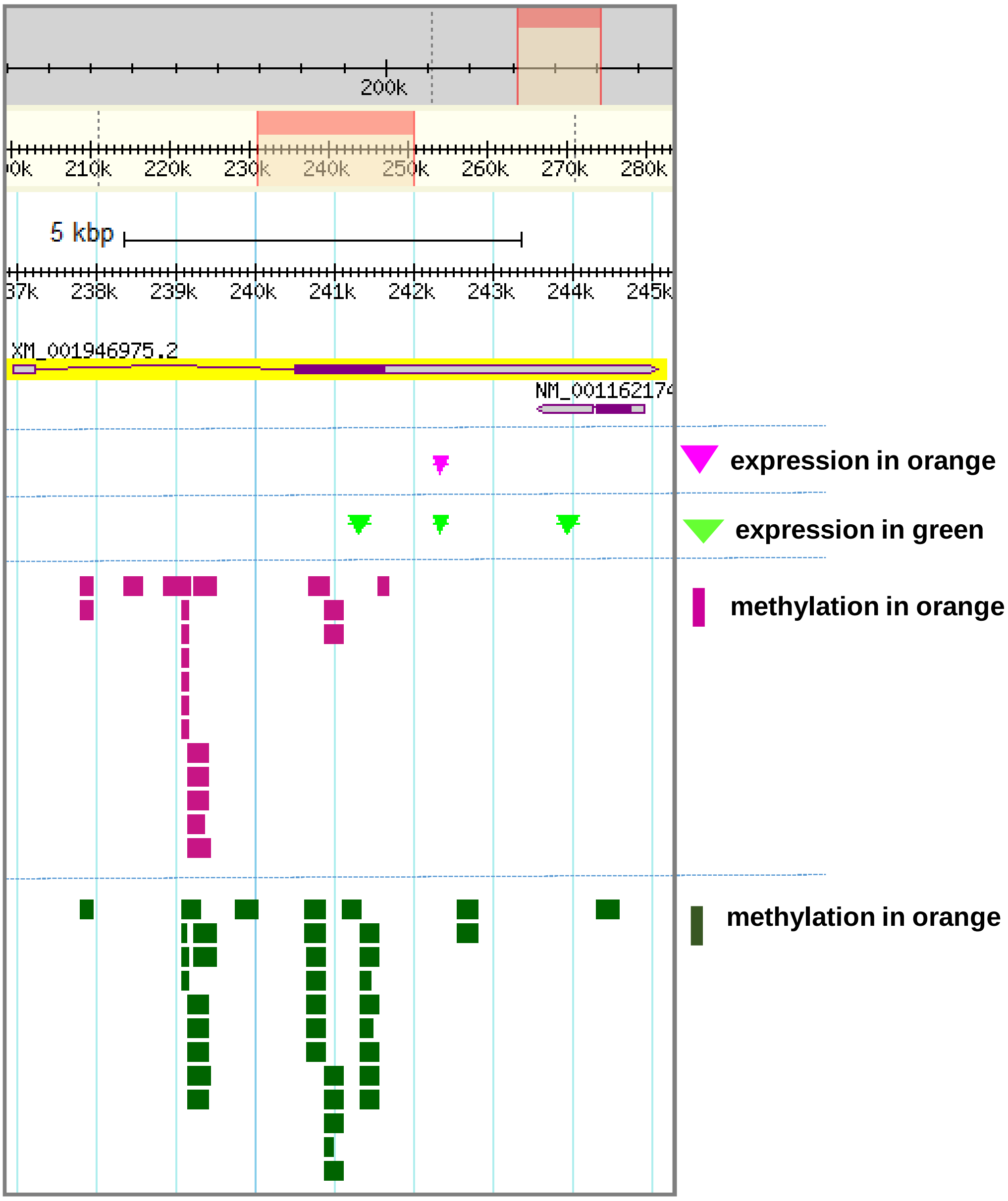

Supplement: S2 Fig — Gbrowse examples showing transcriptomic and methylation data. Representative genes decorates with the mapping of the exact matches corresponding to the methyl reads and the trascriptomic contigs. The reads were directly mapped to the genome scaffolds of A. pisum strain LSR1 version Acryr_2.0. Each pink and green triangles represent a red corresponding to the contigated fragments obtained after subtractive enrichment of transcript. Each pink and green traits represents a methyl read that is at least representing twice in pyrosequencing in the orange and the green. (PDF) [file pone.0115022.s002.pdf]
