# Supplementary material for: Environmentally Selected Aphid Variants in Clonality Context Display Differential Patterns of Methylation in the Genome
Source: PLoS One. 2014 Dec 31;9(12):e115022. doi: 10.1371/journal.pone.0115022 (PMC4281257; doi:10.1371/journal.pone.0115022)
Supplement: S1 Table — List of methyl reads. Lists of the retrieved methylated sequences found in the green and orange samples and the most represented in the genome. These reads match a large number of locations in the genome from 100 to 4500 times. This table is linked to the Fig. 2. (DOCX) [file pone.0115022.s015.docx]

PINK methyl reads: the most represented

>FR3WW0S01AZQJU length=35 xy=289_3736 MID=MID02

ACTTGTAACCTACTGTACAGCAGAGCGACATCCAC

>FR3WW0S01DIC5Y length=50 xy=1322_916 MID=MID02

TAAATTTGAATTCAATGATATAATATCATTGTATAGAAAAACGATTCTGA

>FR3WW0S01AG47D length=75 xy=78_519 MID=MID02

TAATACTATCCATTATACAGTGACCCACTTGTAACCTACTGTACAGCAGAGCGACATCCACTTACCCACCTTTTT

>FR3WW0S01B41PA length=52 xy=760_1868 MID=MID02

CACTTGTAACCTACTGTACAGCAGAGCGACATCCACTTACCCACCTTTTTTA

>FR3WW0S01AZJ78 length=44 xy=287_3734 MID=MID02

ACTTGTAACCTACTGTACAGCAGAGCGACATCCACTTACCCACC

>FR3WW0S01B1E10 length=55 xy=719_486 MID=MID02

TAAATTTGAATTCAATGATATAATATCACTGTATAGAAAAACGATTCTGAGCGGA

>FR3WW0S01BULC9 length=70 xy=641_1563 MID=MID02

TAATACTATCCATTATACAGTGACCCACTTGTAACCTACTGTACAGCAGAGCGACATCCACTTACCCACC

>FR3WW0S01BVANS length=70 xy=649_1574 MID=MID02

TAATACTGTCCATTATACAGTGACCCACTTGTAACCTACTGTACAGCAGAGCGACATCCACTTACCCACC

>FR3WW0S01DV333 length=74 xy=1478_3393 MID=MID02

TAAAAAAGGTGGGTAAGTGGATGTCGCTCTGCTGTACAGTAGGTTACAAGTGGGTCACTGTATAATGGATAGTA

>FR3WW0S01BR2UF length=70 xy=612_3029 MID=MID02

TAATACCATCCATTATACAGTGACCCACTTGTAACCTACTGTACAGCAGAGCGAAATCCACTTACCCACC

>FR3WW0S01CM1XD length=74 xy=965_2287 MID=MID02

TAAAAAAGGTGGGTAAGTGGATGTCGCTCTGCTGTACAGTAGGTTACAAG

TGGGTCACTGTATAATGGATGGTA

>FR3WW0S01CFDE9 length=68 xy=878_291 MID=MID02

TAAGGAAAAACGGGAATTTTTACGCAAAATCTGTTTTCGAGAAAATTGATTTTGGTTTTTGGTGTAAC

>FR3WW0S01AF1FJ length=43 xy=65_2221 MID=MID02

CTTGTAATCTACTGTACAGCAGAGCGACATCCACTTACCCACC

>FR3WW0S01ATNPF length=56 xy=220_2737 MID=MID02

TAAAGTTACACCAAAAACCAAAATCGATTTTCTCGAAAACAGATTTTGCGTAAAAA

>FR3WW0S01EZPI3 length=69 xy=1929_3437 MID=MID02

TAAAGTTACACCAAAAACCAAAATCAATTTTCTCGAAAACAGATTTTGCGTAAAAATTCCCGTTTTTCC

>FR3WW0S01A9ZYU length=71 xy=406_3268 MID=MID02

TAAATACCATCCATTATACAGTGACCCACTTGTAACCTACTGTACAGCAGAGCGACATCCACTTACCCACC

>FR3WW0S01BY0VN length=81 xy=691_3489 MID=MID02

TAATACTATCCATTATACAGTGACCCACTTGTAACCTACTGTACAGCAGAGCGACATCCACTTACCCACCTTTTTTTTTTA

>FR3WW0S01CBK4B length=68 xy=834_3865 MID=MID02

TAAGGAAAAACGGGAATTTTTACGCAAAATCTGTTTTCGAGAAAATCGATTTTGGTTTTTGGTGTAAC

>FR3WW0S01DZ8EX length=68 xy=1525_3079 MID=MID02

TAAGGAAAAACGGGAATTTTTACGCAAAATCTGTTTTCGAGAAAATCGATTTTGGTTTTTGGTGTAAC

>FR3WW0S01AQ7AQ length=79 xy=192_2848 MID=MID02

TAATACCATCCATTATACAGTGACCCACTTGTAACCTACTGTACAGCAGAGCGAAATCCACTTACCCACCTTTTTTTTA

>FR3WW0S01DZ0Q2 length=75 xy=1523_1340 MID=MID02

TAAAAAAAGGTGGGTAAGTGGATGTCGCTCTGCTGTACAGTAGGTTAGAAGTGGGTCACTGTATAATGGACAGTA

>FR3WW0S01E02IY length=59 xy=1945_1400 MID=MID02

TAATCATATCACAATATCCATCAGGTAACGCGTTATACATCAACAACAAACCGTGGTAC

>FR3WW0S01CHECB length=70 xy=901_585 MID=MID02

TAATACTGTCCATTATACAGTGACCCACTTCTAACCTACTGTACAGCAGAGCGACATCCACTTACCCACC

>FR3WW0S01BDNVY length=69 xy=448_2204 MID=MID02

TAAAGTTACACCAAAAACCAAAATCGATTTTCTCGAAAACAGATTTTGCGTAAAAATTCCCGTTTTTCC

>FR3WW0S01CUI0J length=68 xy=1050_2865 MID=MID02

TAAGGAAAAACGGGAATTTTTACACAAAATCTGTTTTCGAGAAAATTGATTTTGGTTTTTGGTGTAAC

>FR3WW0S01CGC21 length=114 xy=889_1451 MID=MID02

TAAATTTGAATTCAATGATATAATATCACTGTATAAGAAAAACGATTCTGAGCGGAGACGGTTTGTCAGTCTAGGTATTAGACATACCTATTATAGGTATACTTATCTATAGTA

>FR3WW0S01CTR6N length=113 xy=1042_861 MID=MID02

TAATACTATAGATAAGTATACCTATAATAGGTATGTCTAATACCTAGACTGACAAACCGTCTCCGCTCAGAATCGTTTTTCTTATACAGTGATATTATATCATTGAATTCAAA

>FR3WW0S01C9B52 length=67 xy=1219_1608 MID=MID02

TAAGGAAAAACGGGAATTTTTACGCAAAATCTGTTTCGAGAAAATTGATTTTGGTTTTTGGTGTAAC

>FR3WW0S01BS4O8 length=76 xy=624_2938 MID=MID02

TAAATTTTTATGAGCGTTTGAAATTCATATTTTTACAACATTTGATATTCACTCGATTTCTTACGTAACGATTTTC

>FR3WW0S01AR4KZ length=74 xy=203_929 MID=MID02

TAAAAAAGGTGGGTAAGTGGATTTCGCTCTGCTGTACAGTAGGTTACAAGTGGGTCACTGTATAATGGATGGTA

>FR3WW0S01CA9VD length=69 xy=831_1575 MID=MID02

TAAAGTTACACCAAAAACCAAAATCAATTTTCTCGAAAACAGATTTTGCGTAAAAATTCCTGTTTTTCC

>FR3WW0S01D1YTI length=107 xy=1545_2036 MID=MID02

TTGTGTCTGTGTACACGATAAGTAGTCGAAATAATGCTACGATTTTCAACTTCAGTATCTTGTTCGATCAGAAAGTGAATATCGTTGGTGCATTGGGGAGGTCAAAA

>FR3WW0S01CYIHO length=68 xy=1096_394 MID=MID02

TAAAGTTACACCAAAACCAAAATCAATTTTCTCGAAAACAGATTTTGCGTAAAAATTCCCGTTTTTCC

>FR3WW0S01DO2IA length=40 xy=1398_2400 MID=MID02

TATAACGCGTTATAAGTACCTAATAGATATTATGATATGA

>FR3WW0S01ELGUS length=72 xy=1767_2562 MID=MID02

AAATTAAGGAAAAACGGGAATTTTTACGCAAAATCGATTTTTCACAAAATTGAATTTGGTTTTTGGTGTAAC

>FR3WW0S01EZ7FS length=69 xy=1935_2070 MID=MID02

TAAAGTTACACCAAAAACCAAAATCAATTTTCTCGAAAACAGACTTTGCGTAAAAATTCCCGTTTTTCC

>FR3WW0S01BPQTJ length=39 xy=586_629 MID=MID02

TAACAAAAGGTGGGTAAGTGGATGTCGCTCTGCTGTACA

>FR3WW0S01EVT10 length=69 xy=1885_2902 MID=MID02

TAAAGTTACACCAAAAACCAAATTCAATTTTGTGAAAAATCGATTTTGCGTAAAAATTCCCGTTTTTCC

>FR3WW0S01D6RNT length=86 xy=1600_759 MID=MID02

TAAATTTTTATGAGCGTTTGAAATTCATATTTTTACAACATTTGATATTCACTCGATTTCTTACGTAACGATTTTCTTATTTTGTT

>FR3WW0S01DTF3L length=117 xy=1448_1839 MID=MID02

TAAATTTTGACCTCCCCAATGCACCAACGATATTCACTTTCTGATCGAACAAGATACTGAAGTTGAAAATCGTAGCATTATTTCGACTACTTATCGTGTACACAGACACAAAAAAAA

>FR3WW0S01EV0IP length=114 xy=1887_3087 MID=MID02

TAAATTTGAATTCAATGATATAATATCACTGTATAAGAAAAACGATTCTGAGCGGAGACGGTTTGTCAGTCTAGTTATTAGACATACATATTATAGGTATACTTATCTATAGTA

Green methyl reads: the most represented

>FR3WW0S01B6S80 length=50 xy=780_2306 MID=MID01

TAAATTTGAATTCAATGATATAATATCATTGTATAGAAAAACGATTCTGA

>FR3WW0S01C2YQW length=53 xy=1146_3286 MID=MID01

TAATACTATCCATTATACAGTGACCCACTTGTAACCTACTGTACAGCAGAGCG

>FR3WW0S01B7WZ9 length=60 xy=793_583 MID=MID01

TAAAAAAAACACACATCATTGTAAAATCAATACATTCATCGTTCCACTCAGAATCTAAAA

>FR3WW0S01BWRGA length=55 xy=666_360 MID=MID01

TAAATTTGAATTCAATGATATAATATCATTGTATAGAAAAACGATTCTGAGCGGA

>FR3WW0S01A1T2L length=62 xy=313_3307 MID=MID01

ACAAACCGTCTCCGCTCAGAATCGTTTTTCTTATACAGTGATATTATATCATTGAATTCAAA

>FR3WW0S01AVDJU length=62 xy=240_968 MID=MID01

TAAAAAAAAAACACACATCATTGTAAAATCAATACATTCATCGTTCCACTCAGAATCTAAAA

>FR3WW0S01BM1R7 length=42 xy=555_1845 MID=MID01

TAATACTATCCATTATACAGTGACCCACTTGTAACCTACTGT

>FR3WW0S01CIKG9 length=70 xy=914_1947 MID=MID01

TAATACTATCCATTATACAGTGACCCACTTGTAACCTACTGTACAGCAGAGCGACATCCACTTACCCACC

>FR3WW0S01C4MH1 length=77 xy=1165_2903 MID=MID01

TAAATTTGAATTCAATGATATAATATCACTGTATAAGAAAAACGATTCTGAGCGGAGACGGTTTGTCAGTCTAGATA

>FR3WW0S01AR0C6 length=38 xy=201_3656 MID=MID01

TAAATTTAGAATTCAATGATATAATATCACTGTATAAG

>FR3WW0S01CTJ5G length=75 xy=1039_2738 MID=MID01

TAAACCTAGACTGACATACCGTCTCCGCTCAGAATCGTTTTTCTTATACAATGATATTATATCATTGAATTCAAA

>FR3WW0S01CYJJI length=68 xy=1096_1756 MID=MID01

TAAGGAAAAACGGGAATTTTTACGCAAAATCTGTTTTCGAGAAAATCGAT

TTTGGTTTTTGGTGTGAC

>FR3WW0S01APFGG length=44 xy=172_2030 MID=MID01

TAAATTTGAATTCAATGATATAATATCACTGTATAAGAAAACGA

>FR3WW0S01A23XH length=60 xy=328_1299 MID=MID01

TAATACTATCCATTATACAGTGACCCACTTGTAACCTACTGTACAGCAGAGTGACATCCA

>FR3WW0S01CQZ31 length=51 xy=1010_2239 MID=MID01

TAAATTTGAATTTCAATGATATAAATATCACTGTATAAGAAAAACGATTCT

>FR3WW0S01ENUQS length=55 xy=1794_3282 MID=MID01

TAAATTTGAATTCAATGATATAATATCACTGTATAGAAAAACGATTCTGAGTGGA

>FR3WW0S01AFFVT length=53 xy=58_2967 MID=MID01

TAAATTTAGATTCTGAGTGGAACGATGAATGTATTGATTTTACAATGATGTGT

>FR3WW0S01ED4Q1 length=61 xy=1684_251 MID=MID01

TAATACTATCCATTATACAGTGACCCACTTGTAACCTACTGTACAGCAGAGCGACATACAC

>FR3WW0S01BL0ER length=79 xy=543_2561 MID=MID01

TAATACTATCCATTATACAGTGACCCACTTGTAACCTACTGTACAGCAGAGCGACATCCACTTACCCACCTTTTTTTCA

>FR3WW0S01ELNVT length=77 xy=1769_3479 MID=MID01

AAGAAAAATTAAGGAAAAACGGGAATTTTTACGCAAAATCTGTTTTCGAGAAAATCGATTTTGGTTTTTGGTGTAAC

>FR3WW0S01ARW7A length=68 xy=200_3652 MID=MID01

TAATACCATCCATTATACAGTGACCCACTTGTAATCTACTGTACAGCAGAGCGACATCCACTTACCCA

>FR3WW0S01ED5AA length=73 xy=1684_944 MID=MID01

TAAAAAGGTGGGTAAGTGGATGTCGCTCTGCTGTACAGTAGGTTACAAGTGGGTCACTGTATAATGGATAGTA

>FR3WW0S01BVHVY length=75 xy=651_2748 MID=MID01

TAAAAAAAGGTGGGTAAGTGGATTTCGCTCTGCTGTACAGTAGGTTACAAGTGGGTCACTGTATAATGGATGGTA

>FR3WW0S01DI1R8 length=70 xy=1330_54 MID=MID01

TAATACCATCCATTATACAGTGACCCACTTGTAACCTACTGTACAGCAGAGCGAAATCCACTTACCCACC

>FR3WW0S01D9PZ7 length=75 xy=1633_3413 MID=MID01

TATAAAAAGGTGGGTAAGTGGATGTCGCTCTGCTGTACAGTAGGTTAGAAGTGGGTCACTGTATAATGGATAGTA

>FR3WW0S01BKIO5 length=68 xy=526_2583 MID=MID01

TAAGGAAAAACGGGAATTTTTACGCAAAATCTGTTTTCGAGAAAATTGATTTTGGTTTTTGGTGTAAC

>FR3WW0S01A10D7 length=62 xy=315_3309 MID=MID01

ACAAACCGTCTCCGCTCAGAATCGTTTTTCTTATACAGTGNTATTATATCATTGAATTCAAA

>FR3WW0S01EA9TW length=70 xy=1651_2034 MID=MID01

TAATACTATCCATTATACAGTGACCCACTTGTAACCTACTGTACAGCAGAGCGACATCCACTTACCCACA

>FR3WW0S01CAQB8 length=54 xy=825_838 MID=MID01

TAAATTTGAATTCAATGATATAATATCATTGTATAAGAAAAAAACGATTCTGAG

>FR3WW0S01CCIA2 length=70 xy=845_1820 MID=MID01

TAATACTATTCATTATACAGTGACCCACTTGTAACCTACTGTACAGCAGAGCGACATCCACTTACCCACC

>FR3WW0S01A8HDO length=80 xy=389_2154 MID=MID01

TAATACTATCCATTATACAGTGACCCACTTGTAACCTACTGTACAGCAGAGCGACATCCACTTACCCACCTTTTTATTTA

>FR3WW0S01ALW5X length=56 xy=132_2195 MID=MID01

TAAAGTTACACCAAAAACCAAAATCGATTTTCTCGAAAACAGATTTTGCGTAAAAA

>FR3WW0S01DE4IC length=69 xy=1285_1282 MID=MID01

TAAAGTTACACCAAAAACCAAAATCAATTTTCTCGAAAACAGATTTTGCGTAAAAATTCCCGTTTTTCC

>FR3WW0S01AKNPH length=73 xy=118_627 MID=MID01

TAATACTATCCATTATACAGTGACCCACTTGTAACCTACTGTACAGCAGAGCGACATCCACTTACCCACCTTA

>FR3WW0S01DXHQU length=70 xy=1494_2180 MID=MID01

TAATACCATCCATTATACAGTGACTCACTTGTAACCTACTGTACAGCAGAGCGACATCCACTTACCCACC

>FR3WW0S01BG9GD length=68 xy=489_2187 MID=MID01

TAAGGAAAAACGGGAATTTTTACGCAAAATCTGTTTTCGAGAAAATCGATTTTGGTTTTTGGTGTAAC

>FR3WW0S01BA51F length=68 xy=420_449 MID=MID01

TAAGGAAAAACGGGAATTTTTACGCAAAATCTGTTTTCGAGAAAATCGATTTTGGTTTTTGGTGTAAC

>FR3WW0S01AQNHZ length=79 xy=186_1765 MID=MID01

TAATACCATCCATTATACAGTGACCCACTTGTAACCTACTGTACAGCAGAGCGAAATCCACTTACCCACCTTTTTTTTA

>FR3WW0S01C1BKD length=79 xy=1128_315 MID=MID01

TAATACTATCCATTATACAGTGACCCACTTGTAACCTACTGTACAGCAGAGCGACATCCACTTACCCACCTTTTTCATA

>FR3WW0S01BK9MR length=176 xy=535_625 MID=MID01

TAAGTGGATGTCGCTCTGCTGTACAGTAGGTTACAAGTGGGTCACTGTATAATGGATAGTATTAAATTTGAATTCAATGATATAATATCACTGTATAAGAAAAACGATTCTGAGCGGAGACGGTTTGTCAGTCTAGGTATTAGACATACC

>FR3WW0S01ELI06 length=80 xy=1768_1288 MID=MID01

TAATACCATCCATTATACAGTGACCCACTTGTAACCTACTGTACAGCAGAGCGAAATCCACTTACCCACCTTTTTTTATT

>FR3WW0S01ARNDI length=70 xy=197_3204 MID=MID01

TAATACTGTCCATTATACAGTGACCCACTTCTAACCTACTGTACAGCAGAGCGACATCCACTTACCCACC

>FR3WW0S01B6KOM length=68 xy=777_3492 MID=MID01

TAAAGTTACACCAAAAACCAAAATCGATTTTCTCGAAAACAGATTTTGCGTAAAAATTCCCGTTTTTC

>FR3WW0S01DHK94 length=58 xy=1313_1642 MID=MID01

TAACTCAAAATAATTTGCTAATTTTCGTGATTTTTCCATATTTTGTCAATTTTTGAAC

>FR3WW0S01ENOWI length=55 xy=1792_3904 MID=MID01

TAAATTTGAATTCAATGATATAATATCATTGTATAGAAAAACGATTCTGANCGGA

>FR3WW0S01DPFCU length=69 xy=1402_2668 MID=MID01

TAAAGTTACACCAAAAACCAAAATCGATTTTCTCGAAAACAGATTTTGCGTAAAAATTCCCGTTTT
