# Supplementary material for: Environmentally Selected Aphid Variants in Clonality Context Display Differential Patterns of Methylation in the Genome
Source: PLoS One. 2014 Dec 31;9(12):e115022. doi: 10.1371/journal.pone.0115022 (PMC4281257; doi:10.1371/journal.pone.0115022)
Supplement: S1 Procedures — Phenotypic selection, demography analysis and detailed molecular biology methods used in this report. (DOCX) [file pone.0115022.s019.docx]

**Supplemental procedures**

**Maintenance and propagation of aphids**

The pea aphid *Acyrthosiphon pisum* belongs to the order Homoptera (Aphididae family) and feeds on the *Vicia faba* plant. Aphids were maintained on *V. faba* in cages in an incubation room at about 22°C +/-3°C and a light/dark photoperiodicity of 16/8 hours. To maintain a clonal population of aphids, apterate adults were collected and placed individually on a new plant in a cage with a mesh siding. The experiments reported herein commenced with a single parthenogenetic founder placed on a *V. faba* plant. This founder was then removed after two days in order to synchronize the progeny. The adults (sisters born from the same mother) were collected simultaneously and placed in a large cage with host plants to further propagate the colony. One adult of this synchronized colony was then placed in a cage/plant system under the desired experimental conditions as indicated in the figures. Aphids were raised at 8°C to select a predominant phenotype (green body color). For this purpose, several cages were placed in a ‘versatile environmental test chamber’ (Sanyo), which was specially engineered for the control of temperature, photoperiodicity and humidity (8°C, 16 h light/8 h dark, 60% humidity).

**Phenotypic selection, extract injection and demography analysis**

Each day, 5 to 10 orange adult aphids raised at 22°C and from a single founder were placed at 8°C as described above. After five months a stable and robust green colony emerged. For the injection experiments, the bacteriome of 20 green adult aphids was roughly excised from the abdomen and ground cautiously in a glass Potter in 500 µl of Ringer solution (115 mM NaCl, 3.5 mM KCl, 2.5 mM NaHCO_3_ and 4.5 mM CaCl_2_). The extract was briefly centrifuged at 1,000 rpm at 4°C for 1 min, and the supernatant was resuspended in the same buffer before injection (1 µg/10 µl). This crude enriched aphid extract was examined by PCR analysis for the methylase *Dam,* the IS4 family transposase and the ribosomal light protein 7 house keeping gene, attesting the presence of endosymbiont bacteria. This soluble extract was then injected in *orange* adult aphids using a micro-syringe under the microscope (magnification 10x) to deliver 10 to 20 µl (about few hundred cells). The same operation was carried out with the *orange* aphid extract injected into *green* and/or *orange* aphids as control. For demography analysis, progenies (from larval stages 1 to 4) were counted each week during one month. The counting of progenies of eight separate experiments was added and the total number divided by the number of initial viable adults that generated these progenies. The control corresponds to the *green* aphids raised at 8°C and was the reference for comparative measures obtained for the three scenarios: passage *green* to *orange*, passage *orange* to *green* and *orange* aphid injected with *green* extract.

**Methyl-collector method for methyl DNA fragment enrichment**

Total DNA was extracted from *A. pisum* and fragmented by *MseI* digestion, which leaves the CpG motif intact. The fragmented DNA was then affinity purified using the MethylCollector kit (Active Motif), based on the recombinant protein containing a methyl binding domain and a 6xHis tag. A protocol provided by the manufacturer allows the isolation of heavily methylated fragments. In previous studies, this procedure was used successfully at a lower scale of experiments (Dombrovsky, 2009). Following methyl affinity precipitation, the resulting DNA fragments were linked with code bar adaptors in order to perform the pyrosequencing step (Coulter Counter/Beckman, Grenoble, France). Five individual preparations were pooled for each phenotype (*green* and *orange*).

**Bisulfite genomic sequencing**

To obtain high throughput and optimal bisulfite genomic sequencing, the commercial kit Methyl Detector (Active Motif) was used. In general, 2 µg aliquots of *A. pisum* gDNA were used for each bisulfite reaction in 10 separate tubes. The reaction conditions were performed to achieve the maximum conversion of unmethylated cytosines to uracil (94ºC for 3 minutes, then 50ºC for 9 hours). The 10 samples of modified bisulfite gDNA were then pooled and served as a template for PCR amplification using specific primers. All the PCR products (2-3 repeats for each gDNA amplified fragment) were purified (Qiagen) and cloned into the pGEM-T-easy vector (Promega). The plasmids were then transformed into DH10B (Invitrogen) competent cells and 20 to 25 independent colonies were used for plasmid purification and sequencing. The percentage of methylation was determined for each site in the PCR fragments. About 50 different genomic sequences were analyzed by this protocol.

**cDNA synthesis and suppression subtractive hybridization**

The synthesis of cDNA was followed by the Suppression Subtractive Hybridization (SSH) procedure. Cogenics Genome Express carried out the pyrosequencing and the analysis of the sequences. The SSH experiment was carried out by using the PCR-Select™ cDNA Subtraction kit from Clontech. According to the manufacturer‘s recommendations, 1.8 µg of each RNA sample were used to synthesize double stranded cDNA. With the aim to identify specific transcripts from the two samples (*green* and *orange* aphid), two SSH reactions were performed by considering each sample either as driver or tester sample. The obtained specific amplicons were purified by using the MinElute PCR purification kit (Qiagen), quantified by Fluorescent measurement with PicoGreen (Invitrogen) and analyzed with a DNA 7500 LabChip on a BioAnalyzer (Agilent). The initial step combines the addition of RNA of 5 individual preparations for the *green* and *orange* aphids.

**Single stranded DNA library**

The same cDNA synthesis protocol was used to create a single stranded (sst) DNA library from genomic or SSH products. According to the GS pyrosequencing protocol, DNA must first be transformed into a library of single-strand template DNA fragments (sstDNA) flanked with amplification and sequencing primer sequences. These sstDNA libraries were prepared using the GS Library Preparation kit (Roche Diagnostics GmbH) according to manufacturer’s recommendations. Each sample is individually treated. Briefly, fragment ends were firstly made blunt before the ligation of the adaptors « A » and « B » allowing for both amplification and sequencing. Multiplex Identifier (MID) was used for identifying samples after pooling them during the pyrosequencing process. To purify sstDNA integrating both the A and the B adaptors, the ligation mixture was immobilized onto magnetic streptavidin-coated beads, via the biotin moiety of Adaptor B. After several wash steps eliminating the unbounded fragments without B adaptors, the sstDNA integrating both the A and B adaptors were melted away from the beads and purified. The quality of the sstDNA was checked by a size range analysis with an RNA 6000 Pico-Assay on the 2100 Bioanalyzer (Agilent Technologies) and the quantification of the sstDNA libraries was carried out by a sensitive fluorescent measurement using the Quant-it™ Ribogreen® RNA assay (Invitrogen).

**emPCR**

Emulsion PCR (emPCR) corresponds to a clonal amplification of the sstDNA library generated previously. For sstDNA library sequencing application, the emPCR is carried out with the GS emPCR Kit I (Roche Diagnostics GmbH). The two genomic samples and the two SSH samples were respectively pooled together. Sixteen and 8 emulsion amplification reactions were prepared for the genomic and the SSH samples, respectively. Briefly, the sstDNA was immobilized onto DNA Capture Beads. The obtained captured DNA library was added to a mixture of amplification mix and oil and vigorously shaken on a Tissue lyser (Qiagen) to create “micro-reactors” containing both the amplification mix and a single bead. Emulsion was dispatched in a 96 well plate and the PCR amplification program was performed according to the manufacturer’s recommendations. After the amplification step, the emulsion was chemically broken and the beads carrying the amplified DNA library were recovered and washed by filtration. Positive beads were purified by means of the biotinylated primer “A” that binds to streptavidin coated magnetic beads. The DNA library beads were then separated from the magnetic beads by melting away the double-stranded amplification products leaving a population of bead-bound single-stranded template DNA fragments. Next, the sequencing primer was annealed to the amplified sstDNA. Lastly, the number of beads carrying amplified sstDNA was evaluated with a Z2™ Cell Counter (Beckman Coulter).

**Sequencing run**

The two Genomic samples and the two SSH samples were simultaneous sequenced in one region of the GS-FLX run using the large bead-loading gasket. According to manufacturer’s recommendations, the right number of DNA beads was loaded per region, followed by the appropriated volume of packing beads and enzyme beads. After the Pre-Wash run, the sequencing run was launched with the “Full Analysis” parameter set. Regarding the obtained reads, the variance was measured under the assumption and observation that many transcripts were not differentially expressed and a large number of methyl genomic reads matching the same genomic position (overlapping or not) was not significantly divergent between the two phenotypes (*green* and *orange*). Although the experimental procedure was carried out using one pyrosequencing step for the two combined phenotypes and two sets of bar codes adaptors (one for the *green* and the other for the *orange*), the starting material was the accumulation of five independent experiments. Consequently we took account of the sequence-specific bias (like those introduced during the library preparation) by a statistical estimation of numbers in order to assess the validity and the robustness of the used protocol.

**Methylome analysis**

The multiplex barcoded pyrosequencing was performed in a single run, with two samples tagged uniquely by multiplex identifiers (MID). The raw data comprised 308,121 reads for a total length of 52,852,594 bp. The reads ranged from 37 bp to 377 bp with an average length of 172 bp. Among these reads, 2,151 (0.7%) didn’t have a recognizable MID and can't be assigned to a sample. 184,082 reads were assigned to the *green* sample and 121,888 reads to the *orange* sample. The reads were directly mapped to the genome scaffolds of the *A. pisum* strain LSR1 version Acyr_1.0 with the program Razers ([Weese et al., 2009](#_ENREF_61)). We set the default mismatch cutoff at 4% so that about 95% of reads can be covered ([Niu et al., 2010](#_ENREF_41)). 77,806 reads from the *orange* sample (63.8%) were mapped to the genome with 65,604 of them (84.3%) mapping to unique genomic regions. For the *green* sample, 123,212 reads (66.9%) were mapped to the genome with 103,529 of them (56.2%) uniquely mapping. The ambiguous reads have 96% identity or more with up to 4,550 different genomic regions (with an average of 40 different regions) for the *green* sample and with up to 6,425 different genomic regions (average 40) for the *orange* sample. Using the same strategy, we aligned the reads to all bacterial genomes available at NCBI. 5,032 reads from the *orange* sample (4.1%) were mapped to a bacterial genome with 4,602 of them aligning to the genome of *B. aphidicola*. For the *green* sample, 12,896 reads (7%) were mapped with 12,426 of them aligning to the *B. aphidicola* genome. All except for one of them were mapped to a unique position. Two reads from the *orange* sample and eight reads from the *green* one mapped to both *A. pisum* and *B. aphidicola*.

**Transcriptome analysis**

We obtained 115,049 transcriptomic reads for a total length of 28,750,667 bp. The reads ranged from 37 bp to 385 bp with an average length of 250 bp. 262 reads (0.23%) were not assigned to a sample, 60,945 reads were assigned to the *orange* sample and 53,842 to the *green* sample. The reads for both samples were assembled using the Newbler 2,0 program ([Margulies et al., 2005](#_ENREF_34)) after the removal of adapter sequences. After the assembly of the sequences, 3,722 contigs were obtained for the *orange* sample and 3,061 contigs for the *green* sample. All singletons were discarded. The contigs were then searched for sequence similarity using BLAST ([Altschul et al., 1990](#_ENREF_1)) against the genome scaffolds of *A. pisum* strain LSR1 version Acyr_1.0. As conclusion, 2,008 contigs from the *green* sample (65.6%) and 2540 contigs from the *orange* sample (68.2%) could be mapped unambiguously to the genome with default BLAST parameters and e-values ≤ 10-50 or better. These contigs were selected for further analysis. The same search for sequence similarity against the genome of *B. aphidicola* results in 22 and 8 reads mapped unambiguously from respectively the *orange* and the *green* sample. None of the contigs were present in both the *A. pisum* and *B. aphidicola* genomes.

**Gene Ontology enrichment**

Methylome reads and transcriptome contigs were aligned to all known and predicted genes of *A. pisum*. A list of genes that show an increase of their expression value in the *green* or the *orange* sample (the level is indicated in figures) has been selected for Gene Ontology term enrichment analysis. From this list of genes, two sublists were created: a list of genes that show a twofold increase of methylation and a list of genes that have a twofold decrease of methylation. The software Gene Ontology Enrichment Analysis Software Toolkit (GOEAST) ([Zheng and Wang, 2008](#_ENREF_66)) was used to test the GO term enrichment within these given gene lists. Two ratios were compared in order to assess the probability of coherent changes: the ratio between the total number of genes found in the aphid genome by automatic annotation (N) and the number of these genes annotated by a given GO term (m) and, on the other side, the ratio between the number of genes in the list (n) and the number of these genes annotated by the GO term (k). The statistical method used to identify significantly enriched GO terms among the lists of genes is the hypergeometric test ([Zheng and Wang, 2008](#_ENREF_66)). A p-value is provided to determine the degree of significance of the GO terms in the list.

**Bioinformatic** **softwares**

The nucleotide sequences were analyzed using a number of bioinformatics programs for promoter prediction:

McPromoter 006 (<http://tools.igsp.duke.edu/generegulation/McPromoter/>,)

MethPrimer (<http://www.urogene.org/methprimer/index1.html>),

PROSCAN ([http://www-bimas.cit.nih.gov/molbio/proscan/](http://www-bimas.cit.nih.gov/molbio/proscan/))

and Promoter 2.0 Prediction Server (<http://www.cbs.dtu.dk/services/Promoter/>).

And reads mapping on genome : Razers

**Supplemental information about the experimental strategies used in this report**

Bisulfite treatment transforms cytosine to uracil whereas methylcytosine stays unmodified. Software analysis will replace U by C and unmodified C means original methy C. In fact bisulfite is a powerful nucleophilic agent which attacks pyrimidine bases (uracil, cytosine and thymine) and not only cytosine although this later is a lot more reactive. Consecutively to bisulfite nucleophilic attack, the cytosine sulfonate derivatives (usually at C6) are very reactive intermediates and will undergo secondary reactions: the most predominant being the induced transamination of the cytosine sulfonate in C4 with whatever nucleophilic groups is in front of, like any amino group of other nucleotides. One other secondary reaction will be an electrophilic substitution at C5 of the intermediate cytosine sulfonate. The sulfonate intermediate is removed by alkali treatment to generate an uracil but another scenario is also the hydrolysis of the base facilitated by the sulfonate, leading to the break down of DNA backbone. The PCR reaction will sort out the “good” templates, leaving the damaged DNA without any way to investigate it. The bisulfite technique appears biased to determine the methylation patterning of the full genome reflecting probably a partial landscape of methylation with no way presently to investigate properly the damaged DNA.

Taken in account these limitations we took another option which consists in performing the pull down of hyper methylated fragments with the restriction of not attributing individual methyl sites. The pull down of heavily methylated fragments (generated by enzymatic cleavage) was performed using an engineered repressor with a strong affinity for the methyl CG (Kd: about 10 ^-9^ M). This technique is not destructive, will not give the exact place of the methyl group on the individual cytosine but allowed us to isolate extensively and reliably the heavily methylated fragments. This is as far as possible than we can reach in term of robustness. To check the pyrosequencing data of these methylated fragments, few specific sequences were analyzed by PCR reaction on the genome treated by bisulfite. These PCR products are inserted in plasmid grown in bacteria and twenty clones for each were sequenced. In such case, bisulfite sequencing will provide in some extent a statistic evaluation of the methylation status site by site and also will constitute an appropriate control of the quality of the pull down.

Methylation data have been calibrated with the huge number of methyl fragments which are unchanged between the variants. Based on these determinations, statistics are consecutively presented on Gene Ontology Enrichment which associates methylation status and variation of expression. Because the phenotypes are “clear cut” we did not conduct RNA seq on the total RNA. RNA seq gives huge amount of information on differences of isoforms, splice junctions and of a large amount of other subtle variations. As we tried to retrieve genes with “on/off ” mode of expression fitting with our “ clear cut ” phenotypes, we proceed with an alternative method which consists to isolate differentially expressed transcripts: the suppression subtractive hybridization (SSH). For these reasons we did differential library from RNA and we pooled samples before pyrosequencing in one run for both samples. In theory this protocol provides an elimination of what is common and enriches what is unique expression of genes in one or the other variant. We used the contigated unchanged reads as internal controls to calibrate the RNA samples (cDNA presenting little changes as background noise related to the PCR linear amplification which escapes to the suppression step at the opposite of PCR exponential amplification observed for the differentially expressed transcripts). See the illustration of the suppression subtractive hybridization (SSH) method below and the description of the exponential and linear PCR amplifications.

Illustration **of the suppression subtractive hybridization (SSH)** (provided by the company )
