# Supplementary material for: Environmentally Selected Aphid Variants in Clonality Context Display Differential Patterns of Methylation in the Genome
Source: PLoS One. 2014 Dec 31;9(12):e115022. doi: 10.1371/journal.pone.0115022 (PMC4281257; doi:10.1371/journal.pone.0115022)

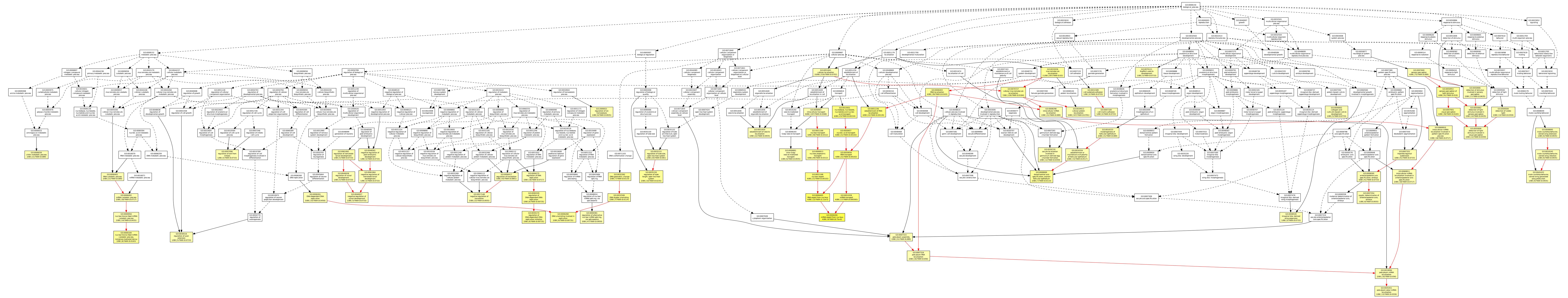

Supplement: S3 Fig — Gene Ontology term enrichment for genes showing a strong decrease in expression: hierarchical relationships in “Biological process”. Selected genes are associated with at least 20 transcriptomic reads in one sample and no reads in the other sample. The graph represents GO term enrichment for the 88 genes for which expression decreases in the green compared to the orange aphids and displays their hierarchical relationships in “Biological process” GO category. Yellow boxes represent GO terms that are significantly enriched, with a p-value <0.1. The degree of color saturation of each node is positively correlated with the significance of enrichment of the corresponding GO term. Non-significant GO terms within the hierarchical tree are drawn as white boxes. Branches of the GO hierarchical tree without significant enriched GO terms are not shown. Edges stand for connections between different GO terms. Red edges stand for relationships between two enriched GO terms, black solid edges stand for relationships between enriched and non-enriched terms, black dashed edges stand for relationship between two un-enriched GO terms. (PDF) [file pone.0115022.s003.pdf]
